# Supplementary material for: Interpreting tree ensemble machine learning models with endoR
Source: PLoS Comput Biol. 2022 Dec 14;18(12):e1010714. doi: 10.1371/journal.pcbi.1010714 (PMC9797088; doi:10.1371/journal.pcbi.1010714)
Supplement: S1 Text — (PDF) [file pcbi.1010714.s001.pdf]

Interpreting tree ensemble machine learning models with endoR - S1 Text

Albane Ruaud[a], Niklas Pfister[b], Ruth E Ley[a], Nicholas D Youngblut[a,*]

[a]Max Planck Institute for Developmental Biology, Department of Microbiome Science, Tuebingen, Germany
[b]University of Copenhagen, Department of Mathematical Sciences, Copenhagen, Denmark
* nicholas.youngblut@tuebingen.mpg.de

Supplementary Methods: description of endoR

Here, we describe methodological and technical procedures in more details when needed compared to the main text.

Rules, decisions and decision ensembles. For a fixed decision D and a variable x^j, or pair of variables $\{x^j, x^k\}$, the *complement decision* D^{c}_j, or $D^{\mathrm{c}}_{j,k}$, are defined to be the decisions resulting from modifying rule r_D to have the complement support for the variable x^j, or the pair of variables $\{x^j, x^k\}$ (S16 Fig), i.e.,

$$r_{D^{\mathrm{c}}_j}(\mathbf{x}) := \mathbb{1}_{\mathbb{R}\setminus\mathcal{X}^j_{r_D}}(x^j)\prod_{k\neq j}\mathbb{1}_{\mathcal{X}^k_{r_D}}(x^k)$$

$$\text{or } r_{D^{\mathrm{c}}_{j,k}}(\mathbf{x}) := \mathbb{1}_{\mathbb{R}\setminus\mathcal{X}^j_{r_D}}(x^j)\mathbb{1}_{\mathbb{R}\setminus\mathcal{X}^k_{r_D}}(x^k)\prod_{l\notin\{j,k\}}\mathbb{1}_{\mathcal{X}^l_{r_D}}(x^l),$$

respectively.
Additionally, decisions D^{rm}_j and $D^{\mathrm{rm}}_{j,k}$ are defined to be the decisions resulting from removing the variable x^j, or pair of variables $\{x^j, x^k\}$, from the rule r_D (Figure 15), i.e.,

$$r_{D^{\mathrm{rm}}_j}(\mathbf{x}) := \prod_{k\neq j}\mathbb{1}_{\mathcal{X}^k_{r_D}}(x^k) \quad\text{and}\quad r_{D^{\mathrm{rm}}_{j,k}}(\mathbf{x}) := \prod_{l\notin\{j,k\}}\mathbb{1}_{\mathcal{X}^l_{r_D}}(x^l),$$

respectively.
Finally, for a subset of variables $J\subset\{x^j, j\in\{1,\ldots,p\}\}$ the decision D^{pr}_J is defined to be the decision resulting from removing all variables not included in J from r_D, i.e.,

$$r_{D^{\mathrm{pr}}_J}(\mathbf{x}) := \prod_{k\in J}\mathbb{1}_{\mathcal{X}^k_{r_D}}(x^k).$$

The predictions $\hat{y}_{D^{\mathrm{c}}_j}$, $\hat{y}_{D^{\mathrm{c}}_{j,k}}$, $\hat{y}_{D^{\mathrm{rm}}_j}$, $\hat{y}_{D^{\mathrm{rm}}_{j,k}}$ and $\hat{y}_{D^{\mathrm{pr}}_J}$ are each updated based on the new rule.

For a variable x^j, we define the set of *active decisions* as $\mathcal{D}^j := \{D\in\mathcal{D} | \mathcal{X}^j_{r_D}\neq\mathbb{R}\}$, the subset of decisions which depend on x^j. Likewise, the set of active decisions of a pair of variables $\{x^j, x^k\}$ is defined as $\mathcal{D}^{j,k} := \mathcal{D}^j\cap\mathcal{D}^k$.

Extraction of rules from predictive models. Decisions are extracted from tree-based models (randomForest, ranger, gbm and xgboost (1–4)) using the inTrees R-package (5), with slight modifications. More specifically, given a tree-based model, rules are first extracted from all trees, or a subset of them, by following branches from the root down to the terminal node, e.g., for a tree composed of 4 terminal nodes, 4 decisions would be extracted.
All multi-class factor predictive variables are converted to $\{0,1\}$ encoded dummy variables. Extracted rules are then adjusted to be using only one class of each of the original multi-class factor variables and rules multiplicity is decreased accordingly. For instance, for a multi-class factor x^j taking values in $\{a,b,c\}$, three dummy variables would replace x^j and a rule such as "$x^j\in\{a,b\}$" would be transformed into two rules "$x^j_a = 1$" and "$x^j_b = 1$" with multiplicity equal to 0.5. In addition, the same procedure of rule splitting is applied to predictive factors provided by users, that were already encoded as dummy variables for fitting the predictive model. Levels of multivariate variables are thus included only by their presence, later helping with the visualization and interpretation of networks.

Rational for the expression of the interaction importance. Consider the decision D with rule "$x_1 > 0$ & $x_2 > 0$" and prediction "y". From the classical statistical standpoint the interaction between "$x_1 > 0$" and "$x_2 > 0$" is important if the same prediction can not be achieved by using only "$x_1 > 0$" or only "$x_2 > 0$". To assess this we look at the following two modified decisions:

1. D^{rm}_1: rule "$x_1 > 0$ & $x_2 > 0$" and prediction "y_1" (where y_1 is based on rule "$x_2 > 0$")
2. D^{rm}_2: rule "$x_1 > 0$ & $x_2 > 0$" and prediction "y_2" (where y_2 is based on rule "$x_1 > 0$")

D_1^{rm} corresponds to the best prediction (on the same subselection of the data "$x_1 > 0$ & $x_2 > 0$") that a decision only using "$x_2 > 0$" can achieve. Similarly, D_2^{rm} corresponds to the best prediction (on the same subselection of the data "$x_1 > 0$ & $x_2 > 0$") that a decision only using "$x_1 > 0$" can achieve. We can now compare the predictive performance of the different rules, which is done by looking at the decision-wise feature importance. I.e., if D_1^{rm} is better than D the decision-wise importance of x_1 (d_1) is large and if D_2^{rm} is better than D the decision-wise feature importance of x_2 (d_2) is large. Now we can consider the following combinations:

- "d_1 large and d_2 large": This indicates that there is an interaction effect because dropping either variable leads to a decrease in performance. And indeed the decision-wise feature interaction will be large.
- "d_1 small and d_2 large": This indicates that only "$x_2 > 0$" is important implying that there is at most a small interaction effect. In this case the decision-wise feature interaction will be small.
- "d_1 large and d_2 small": This indicates that only "$x_1 > 0$" is important implying that there is at most a small interaction effect. In this case the decision-wise feature interaction will be small.
- "d_1 small and d_2 small": Neither "$x_1 > 0$" nor "$x_2 > 0$" are important in this case and therefore the interaction effect should also be small.

Note that the square root additionally ensures that the feature and interaction importances remain on the same scale.

Feature and interaction direction. To understand how a single feature influences the prediction, one needs to understand whether a rule uses predominantly small or large values of that feature. For every decision D and variable x^j, the *direction indicator* $d_D^j \in \{-1,1\}$

$$d_D^j := \begin{cases} 1 & \text{if } \frac{1}{|S_D|}\sum_{i\in S_D} x_i^j \geq \frac{1}{|S_{D_j^c}|}\sum_{i\in S_{D_j^c}} x_i^j \\ -1 & \text{if } \frac{1}{|S_D|}\sum_{i\in S_D} x_i^j < \frac{1}{|S_{D_j^c}|}\sum_{i\in S_{D_j^c}} x_i^j \end{cases}$$

expresses whether D predominantly uses small or large values of variable x^j.
For every pair of variables $\{x^j, x^k\}$,

$$\eta_{j,k} := \mathrm{sign}\Big(\sum_{D\in\mathcal{D}^{j,k}} (d_D^j \cdot d_D^k \cdot I_D)\Big)$$

records whether variables $\{x^j, x^k\}$ are each associated with y in the same direction across $D \in \mathcal{D}^{j,k}$. When both variables $\{x^j, x^k\}$ have large, or small, values associated with the response y, then $\eta_{j,k}$ is positive; and when large values of x^j are positively associated with y but small values are positively associated with y, then $\eta_{j,k}$ is negative. The later occurs when $\gamma_D^{j,k} = 0$.

Regularization of the decision ensemble. We propose several procedures to regularize the decision ensemble and so reduce the noise by including a simplicity bias. These procedures are recommended but optional.

Decision discretization: quantiles of variable distribution Numerical predictors can be discretized based on their quantiles (e.g., into levels 'Low', 'Medium' and 'High'). All decisions containing discretized variables are then modified by replacing any numeric rule (e.g., '$x^j \leq t$') by the best approximating rule which only uses the discretized variables (e.g., ' x^j ='Low' '). Decisions consisting of the same rules are grouped, the multiplicity is recorded, i.e., how many decisions have been collapsed into the simplified decision) and the prediction, error, support and importances are re-computed based on the updated rule, and the decision importance is weighted by the decision multiplicity. Finally, the feature influence is computed for each level of discretized variables and the feature importance is calculated across all levels.
In practice, all, or a user-defined subset of, numeric variables are discretized based on their quantiles using the `discretizeVector` function from the inTrees R-package (5), adapted to accept missing values (NA). For each rule containing discretized variable, numeric thresholds are replaced by corresponding levels for which the majority of observations are included in the original sample support (S8 Fig). Rules are then transformed as described in the above section to be based on only one level, and the multiplicity is updated.

Decision discretization: local maxima of tree ensemble model splits Alternatively, we propose to discretize numeric variables based on their use in the predictive model. For each numeric variable, we first collect all thresholds of splits on this variable in the model. All thresholds outside of the variable range are given the maximal or minimal values of the variable, i.e., for a threshold t from a split on variable V, if $t > max(V)$, $t \leftarrow max(V_i)$ or if $t < min(V)$, $t \leftarrow min(V)$. Then, we compute their distribution and calculate the local maxima. These maxima are used as limits for the groups of the new discretized variable such that the K-1 greatest maxima are used to make K categories.

Decision pruning Pruning consists of removing variables from decisions that do not participate much to a decision, i.e., for which the difference in errors of the decision with and without the variable is low (5). Comparison of errors can be performed

using the absolute or relative difference in errors (absolute difference by default) (5). Accordingly, the procedure looks for the smallest subset of variables J with the lowest error, such as,

$$\alpha(S_{D_J^{\text{pr}}}, \hat{y}_{D_J^{\text{pr}}}) - \alpha(S_D, \hat{y}_D) \leq \theta \qquad \text{or} \qquad \frac{\alpha(S_{D_J^{\text{pr}}}, \hat{y}_{D_J^{\text{pr}}}) - \alpha(S_D, \hat{y}_D)}{\max\left(\alpha(S_D, \hat{y}_D), 10^{-6}\right)} \leq \theta, \tag{1}$$

with θ a user-specified threshold ($\theta = 0.05$ by default). If Equation Eq. (1) is not satisfied by any J, i.e., for all simplified decisions the differences in error are above the threshold, the original decision is returned. The prediction, error, support, importance and multiplicity are re-computed based on the updated rule, and the decision importance is weighted by the decision multiplicity.

Decision ensemble regularization When bootstrapping is not feasible, we propose to instead filter out all decisions with an importance below a given threshold λ_{imp}, selected by the user or using the following heuristic procedure

$$\lambda_{\text{imp}} := \underset{\lambda}{\text{argmax}} \frac{|\mathcal{D}| - |\mathcal{D}(\lambda)|}{|\mathcal{D}(\lambda)|} \sum_{D \in \mathcal{D}(\lambda)} I_D,$$

where $\mathcal{D}(\lambda)$ is the set of decisions with $I_D \geq \lambda$.

Constructing the network. After regularization and computing all metrics, we propose to visualize the feature and interaction importance and influence in a network. In particular, nodes in the network correspond to single variables and edges to interactions between variables. More specifically, for every node $j \in \{1, \ldots, p\}$, we choose the node size and color in the following way:

- *node size:* feature importance F^j. Larger nodes correspond to more important variables;
- *node color:* feature influence Γ_j, where the color interpolates from blue to orange (via white), with blue corresponding to small prediction values, white to prediction values close to the mean response variable across all samples, and orange to large prediction values.

Similarly, for every pair of nodes $\{j, k\} \in \{1, \ldots, p\}^2$, the edge between the two nodes is chosen as follows:

- *edge width:* interaction importance $F_{j,k}$. Thicker edges correspond to more important interactions;
- *edge color:* interaction influence $\Gamma_{j,k}$, with the same color scale than for nodes;
- *edge type:* interaction direction $\eta_{j,k}$. It is either a solid line if the pair of variables is on average used in the same direction in decisions, i.e., they are positively associated, and it is a dashed line otherwise.

The network object is created using the igraph and ggraph R-packages (6, 7), hence being compatible with the broadly employed ggplot2 R-package (8).

Implementation. We implemented the whole method described above, together with functions to visualize results, into an open source R-package available on GitHub (aruaud/endoR).

The main wrapper function of the endoR package takes as inputs (i) a predictive model fitted using the randomForest, ranger, gbm or XGBoost R-packages (1–4), and (ii) data and a response variable on which to fit the decision ensemble, being the ones used to fit the model or not. Upon starting, all factor variables are transformed into dummy variables, and, in the case of multiclass classification, the problem is transformed into a binary classification problem according to the class defined by the user to focus on. All regularization steps, i.e., discretization, pruning, filtering and bootstrapping, are optional and parameters can be elected by the user. The current implementation was optimized using the data.table R-package (9) and can be ran locally in parallel via the parallel R-package (10). Moreover, bootstrapping can be performed in parallel, locally or on a high-performance computing (HPC) environment, with the clusterMQ R-package (11).

Bibliography

1. Andy Liaw, Matthew Wiener, et al. Classification and regression by randomforest. *R news*, 2(3):18–22, 2002.
2. Marvin N. Wright and Andreas Ziegler. ranger : A Fast Implementation of Random Forests for High Dimensional Data in C++ and R. *Journal of Statistical Software*, 77(1), 2017. ISSN 1548-7660. doi: 10.18637/jss.v077.i01.
3. Brandon Greenwell, Bradley Boehmke, Jay Cunningham, and GBM Developers. gbm: Generalized Boosted Regression Models, 2020.
4. Tianqi Chen and Carlos Guestrin. Xgboost: A scalable tree boosting system. In *Proceedings of the 22nd acm sigkdd international conference on knowledge discovery and data mining*, pages 785–794, 2016.
5. Houtao Deng. Interpreting tree ensembles with inTrees. *International Journal of Data Science and Analytics*, 7(4):277–287, June 2019.
6. Gabor Csardi and Tamas Nepusz. The igraph software package for complex network research. *InterJournal*, Complex Sy:1695, 2006.
7. Thomas Lin Pedersen. ggraph: An Implementation of Grammar of Graphics for Graphs and Networks, 2020.
8. Hadley Wickham. *ggplot2: Elegant Graphics for Data Analysis.* Springer-Verlag New York, 2016. ISBN 978-3-319-24277-4.
9. Matt Dowle and Arun Srinivasan. *data.table: Extension of 'data.frame'*, 2020. R package version 1.13.4.
10. R Core Team. *R: A Language and Environment for Statistical Computing.* R Foundation for Statistical Computing, Vienna, Austria, 2020.
11. Michael Schubert. clustermq: Evaluate Function Calls on HPC Schedulers (LSF, SGE, SLURM, PBS/Torque), 2020.
