# Supplementary material for: Interpreting tree ensemble machine learning models with endoR
Source: PLoS Comput Biol. 2022 Dec 14;18(12):e1010714. doi: 10.1371/journal.pcbi.1010714 (PMC9797088; doi:10.1371/journal.pcbi.1010714)
Supplement: S2 Text — (PDF) [file pcbi.1010714.s002.pdf]

Interpreting tree ensemble machine learning models with endoR - S2 Text

Albane Ruaud[a], Niklas Pfister[b], Ruth E Ley[a], Nicholas D Youngblut[a,*]

[a]Max Planck Institute for Developmental Biology, Department of Microbiome Science, Tuebingen, Germany
[b]University of Copenhagen, Department of Mathematical Sciences, Copenhagen, Denmark
* nicholas.youngblut@tuebingen.mpg.de

Supplementary Methods: data, evaluation of endoR, and metagenome analysis

Here, we describe methodological and technical procedures in more details when needed compared to the main text.

Data to evaluate endoR.

Fully simulated data. Sets of simulations were performed with the following data parameters: n = 200, 1000 or 5000 samples and r = 0.05, 0.1 or 0.2 (with $n = 1000$ and $r = 0.05$ unless mentioned). Each set was replicated in 100 independent simulations (Figs 3D-F and S5A-E and G-H), and a single replicate of the data with parameters $n = 1000$ and $r = 0.05$ is given in S4 Fig.
We assessed the efficiency of the method to extract the right information from models by fitting an RF model on each simulation via the randomForest R-package (1) with default parameters. To tweak the accuracy of RF models for a same set of simulations and endoR parameters, we additionally the number of trees in the forest (randomForest parameter ntree = 10, 100 or 500, the default). For the replicate on S4 Fig, we report the average accuracy of models on 10-folds cross-validation (CV) $0.7 - 0.3$ train-test. The accuracy of the model fitted on all data is reported otherwise (Figs 3D-F and S5A-E and G-H, and S1 Table).
Each classifier was processed with endoR using default parameters, $B = 100$ bootstrap resamples with $\alpha = 5$ for the replicate in S4 Fig, and $B = 10$ with $\alpha = 5$ for the replicates in S5 Fig.

Artificial phenotypes. Data consisted of a subset of the metagenomes used in Youngblut et al. (2), so that samples with the following reported information were removed: i) samples from rectal swabs; ii) samples from individuals suffering from mumps, coeliac disease, gestational diabetes, cholera or with high relative abundances of *Vibrio cholerae*, infected by shiga toxin-producing *Escherichia coli* or cytomegalovirus; iii) samples with less than a million of sequence reads; iv) samples with missing age information. In total, metagenomes from 2147 samples from 19 studies and 23 countries were gathered. Microbial relative abundances were generated by Youngblut et al. (2) using a custom database based on the Genome Taxonomy Database (GTDB), Release 89.0 (3), created with the Struo pipeline (4). Only the relative abundances of families, genera and species with a prevalence above 25 % were included ($p = 520$ taxa).
We fitted a model predicting each artificial phenotype with the relative abundances of microbial families, genera and species, along with the multi-class group factor K. Note that multiple taxonomic levels were included to mimic a situation where no prior knowledge on associations between the microbiome composition and phenotype is available. Model fitting consisted of a feature selection step followed by the fitting of the random forest classifier. Feature selection was included in CV to reduce colinearity among predictors, noise and *in fine*, the dimensionality of data. Methods for models fitting are detailed below. The average Cohen's κ of models from 10-folds CV 0.7-0.3 train-test sets are reported in S10 Table.
Each model was processed with endoR using default parameters and $\alpha = 10$. For the main replicate, we used discretization into 3 categories and $B = 100$. For the repetitions on phenotype simulations, we varied B to be 10, 50 or 100 and α to be 1, 5, 10, 15 or 20 (Figs 3A and S5F-J).

Cirrhosis metagenomes. Metadata and gut microbial taxonomic profiles from metagenomes generated by Qin et al. (5) were downloaded from the MLRepo (https://github.com/knights-lab/MLRepo, accessed on 27/01/2021). The data set consisted of 130 stool samples from cirrhotic and healthy individuals, from which gDNA was extracted and sequenced via an Illumina HiSeq sequencer. The metagenomes had been taxonomically profiled with BURST (6) and Prokaryotic RefSeq Genomes. The downloaded taxonomic profiles consisted of read counts for taxonomic levels not collapsed at coarser level (i.e., if a read count had been assigned to the species level, the number of count of the genus was not indicated). Consequently, we calculated the read counts for each taxonomic level by summing read counts of all species in the clade. Relative abundances were then normalized per the total number of reads to obtain relative abundances. Taxa were filtered using a progressive threshold as described below.

Progressive filtering of rare taxa. Taxa with low mean abundance or low prevalence were filtered out if, for a taxa t of prevalence P and average abundance A:

$$P(t) < A(t) \cdot \beta_0 + \beta_1,$$

with:

$$\beta_0 := \frac{\text{median}_{i\in\{1,...,T\}}(P(t_i)) - P_{q1}}{A_{q1} - \text{median}_{i\in\{1,...,T\}}(A(t_i))}$$
$$\beta_1 := P_{q1} - \beta_0 \cdot \text{median}_{i\in\{1,...,T\}}(A(t_i)),$$

and, P_{q1} and A_{q1} corresponding to the prevalence and abundance quantile values for 25% of all taxa. This continuous filtering allows to keep taxa with low abundances but high prevalence, and inversely keep highly abundant taxa present only in a small set of samples.

Feature selection and fitting of models on metagenome data. Microorganisms are named according to a taxonomy that divides them into groups arbitrarily defined and not consistently reflecting metabolic capacities or specificities (7, 8). Consequently, describing microbial diversity with a unique taxonomic level may not capture microbial interactions in a community. Accordingly, here we included relative abundances of the family, genus, and species taxonomic ranks. The subsequent drawback is the high-dimensionality of the data, i.e., the high number of predictive variables p relative to the number of observations n. As p increases, the set of n observations will represent a relatively smaller set of the p-dimensions space (9). It will thus be harder for models to evaluate the general association of each variable or even interactions of variables with the phenotype, and associations detected may be true only for the specific set of samples used for analyses (i.e., the model will overfit data). While RF are more robust to overfitting thanks to the high number of trees built on sample bootstraps, they are more sensitive to the input feature due to the growth of trees on subsets of variables (9, 10). If many irrelevant features are included, the probability to select the true predictive ones will decrease.

Therefore, feature selection (FS) was performed before fitting an RF model to select the most relevant variables. Feature selection algorithms and parameters were evaluated during training: feature selection and fitting of a classifier were performed on training data and a test set was then predicted to evaluate the model performance (S1 Fig). The RF was fitted with default parameter (1). A boosted tree model was alternatively fitted instead of the RF using the XGBoost R-package (default parameters and nrounds = 10) (11). The choice of the feature selection algorithm, classifier, and parameters was determined using 10 CV with a $0.7-0.3$ train-test split of the data: the model that resulted in the highest average Cohen's κ was selected and a final full-model, *i.e.,* feature selection and fitting of classifier, was then refitted to the entire data (Table 1 below).

The types of models considered for the metagenome experiments were the following:

- `randomForest` function from the randomForest R-package (no FS);
- subselect variables using the Boruta R-package (both functions `Boruta` and `TentativeRoughFix` with default parameters) and then apply `randomForest` from the randomForest R-package;
- subselect variables using the gRRF algorithm from the gRRF R-package for values of γ between 0 and 1 and, for each set of features selected with a different γ value, apply `randomForest` from the randomForest R-package;
- subselect variables using a modified version of the gRRF algorithm to take into account the taxonomy (see the following section), for values of γ and of k between 0 and 1 and, for each set of features selected with a different (γ, k) couple, apply `randomForest` from the randomForest R-package.

The choice of the Boruta and gRRF algorithms was motivated by the ability of Boruta to select all relevant variables (12), hence most likely to include all correlated variables, and for the ability of gRRF to select only relevant and non-redundant variables (13). We additionally modified the expression of the regularization term in the gRRF algorithm, to account for the hierarchical taxonomic structure in metagenomes (in the following *Taxa-aware feature selection* section).

Taxa-aware feature selection. Due to the hierarchical structure of nested taxonomic levels, and so their inter-dependency, redundancy occurs when including several taxonomic levels in analyses (14). But, as prior knowledge on which taxonomic levels are the most relevant is limited, it can be delicate to choose which ones to include. Nonetheless, the noise added by the inclusion of several taxonomic levels can be removed by taking the hierarchy of features into account when performing feature selection (8, 15, 16). Here we propose to modify the gRRF feature selection algorithm (13) to consider the taxonomic structure. For this, we simply add a term reflecting the importance of taxa taxonomically related to the focal one i when calculating its regularization term λ_i. Hence, the original λ_i,

$$\lambda_i := 1 - \gamma + \gamma \frac{Imp_i}{Imp*}, \tag{1}$$

becomes

$$\lambda_i := 1 - \gamma + \gamma \Big(\frac{Imp_i}{Imp*}\Big)^{1-k} \Big(\frac{Imp_i}{max(Imp_j | j \in b)}\Big)^k, \tag{2}$$

with b the subset of variables in the same taxonomic branch than variable i. For variables not describing a taxon, e.g., a metadata, λ_i remains calculated according to Equation Eq. (1) and not Eq. (2).

Table 1. Cross-validation (CV) of feature selection and training of classifiers on metagenomic data.

Data	Feature selection[a]	RF accuracy[b] (%)	Cohen's κ[b]	N features[d]
Main replicate of artificial phenotype	None	68.69±0.83	0.36±0.02	525
	**gRRF (γ=0.45)**	**85.19±2.36**	**0.70±0.05**	**18**
	taxa-aware gRRF[c] (γ=0.25, k = 0.25)	73.06±3.19	0.44±0.07	75
	Boruta	77.62±1.44	0.54±0.03	91
Cirrhosis	None	83.42±4.48	0.67±0.09	926
	gRRF (γ=0.1)	86.58±4.88	0.73±0.10	46
	**taxa-aware gRRF[c] (γ=0.9, k = 1)**	**86.58±3.81**	**0.73±0.08**	**69**
	Boruta	85.53±4.68	0.71±0.09	37

[a] The best feature selection selection algorithm is indicated in bold.
[b] Average and standard deviations across CV repetitions.
[c] A range of γ and k were tested for parameter tuning, but for concision, only results for the γ and k resulting in the best model are reported.
[d] Number of selected features for the model fitted on all data.

In the present article, we define b, as all taxa directly up- and downstream the focal one. To the finest taxonomic level used in analyses, we add the sister levels to b. Therefore, since we here included the family, genus and species levels to our analyses, b was defined for each level as:

- family: the family and all its genera;
- genus: the genus, the family it belongs to and all its species;
- species: the genus it belongs to and all species of that genus.

Both γ and k were tuned to evaluate how much weight should be given to gRRF Gini importances and to the taxonomic term in Equation Eq. (2), respectively. For each model sequence, 121 combinations of feature selection parameters were tested. For instance, although including the term improved models accuracy for the prediction of cirrhosis from metagenomes, it did not for the prediction of our simulated target (Table 1 above).

Comparison of endoR with other analysis methods.

Accuracy in identifying relevant variables and interactions of variables. We compared the performance of endoR in identifying true variables and interactions of variables with those of other methods commonly used for metagenome analysis.
The statistical tests used to correlate biological variables to a phenotype often are the Wilcoxon-rank sum and χ^2 tests, for numeric and categorical variables respectively. Those non-parametric tests are preferred over parametric ones due to their looser assumptions on data (e.g., variable distribution). For all numeric variables in the metagenomes (p = 520 taxa) we performed a Wilcoxon-rank sum test to compare taxa relative abundances in target categories against each other, and we performed a χ^2 test to assess whether groups were counting more samples than expected from one or the other target category. All p-values were adjusted with the Benjamini-Hochberg correction method. To assess the performance of non-parametric tests, variables were ordered by increasing adjusted p-values and were sequentially added to calculate the number of TP and FP variables.
Regularized linear models are white-box simple models that have shown to permorm as well as random forests on microbiome data (17). We compared endoR to lasso regularized models (18) generated with the glmnet R-package (19). Data were first transformed using the center-log ratio. The transformation was performed per taxonomic level on relatives abundances of all taxa (i.e., before filtering and on all families, then all genera, then species) to respect the compositionality. Two lasso models were fitted: the first model did not include interactions (i.e., only variable main effects) and the second included all variables and pairwise interactions. The lambda parameter was tuned and models were fitted with the cv.glmnet function, with parameters nfolds = 10 (the number of CV sets), family = 'binomial' (to perform a binary classification), nlambda = 100 (the number of lambdas to try during training), and alpha = 1 (a regularization parameter which results in lasso regularization when set to 1). For each model, variables were ranked based on their absolute weight; for the model with interactions, we used for each variable the sum of its main effect and all interactions weights.
Sparse covariance matrices are used in microbiome science to determine conditionally non-independent taxa and build correlation networks (20). The comparison of networks computed for distinct sample groups allows one to identify different associations in different groups of samples. For instance, by comparing networks extrapolated for samples collected from environment A versus environment B, one can to infer associations of variables specific to each habitat (21). A drawback of covariance matrices is the exclusion of categorical variables from analysis. Hence, for our application to the artificial phenotypes, we could not include the group variable to the analysis. We computed the covariance matrices of samples within each target category and selected all edges not shared between the two sub-networks to estimate the accuracy of the identified edges. For

each pair of variables, the square of the matrix parameter was calculated (to obtain the magnitude of the correlation between variables), pairs of variables were ordered in descending values of the square parameter, and sequentially added to calculate the number of TP and FP. Several methods exist to estimate covariance matrices, with all proposing different approaches to deal with the compositionality and expected sparsity in metagenome data. Here, methods implemented in the SpiecEasi R-package (22) were employed:

- the sparCC algorithm, which calculates the variance among observations of differences in log-transformed pseudocounts of taxa relative abundances, to estimate the covariance matrix (21);
- the (23) algorithm, which fits Lasso models on each pair of variables and uses the estimated penalization parameters to make the covariance matrix;
- the graphical Lasso method (20), which similarly to (23) fits Lasso models and uses the estimated penalization parameters to make the covariance matrix but repeats models fitting such as to maximize the log-likelihood of variables to follow a Gaussian distribution.

Due to the low accuracy of the (23) method on the artificial phenotypes, results are not discussed nor shown in the present paper. This was expected as the (23) method is a simpler approximation of the covariance matrix, as suggested by (20).

Finally, we compared endoR to methods for interpretation of tree-based models. The most straightforward approach was to use the Gini importance (10, 24) from the same RF classifier we used for endoR, as implemented in the randomForest R-package (24). Then, we computed SHAP values (25, 26), as implemented in the iBreakDown R-package (27), with the default number of 25 random paths. SHAP estimations were then averaged across random paths for each sample and variable. For each variable, its absolute values across samples were finally averaged to obtain global SHAP values.

Since implementations of SHAP for RF classifiers in R do not return interaction values (see (27, 28) and the fastshap R-package), we additionally fitted an XGBoost model (11) to the metagenomes and artificial phenotype, with default parameters and $nrounds = 10$. SHAP values and SHAP interaction values were directly extracted from the XGBoost model by setting the `predcontrib` parameter to TRUE when fitting the model. Figures of SHAP values were created using the SHAPforxgboost R-package (29) (S11 Fig). The fitted XGBoost model was also processed with endoR for comparison.

For both SHAP and Gini importance methods, variables were sorted in descending order of feature importance (respectively the SHAP value and Gini importance) and sequentially added to calculate the number of TP and FP.

Computation time. We measured the computation time and memory needed to run endoR on a single replicate of the artificial phenotype data; results were compared to the `shap` function from the iBreakDown R-package (27) for comparison (S7 Fig and S11 Table). We focused on RF for comparison with SHAP, as SHAP can be directly extracted from an XGBoost model (11), hence not requiring any additional processing time. Runs were performed in triplicates for the measurement of the total CPU time and maximal virtual memory used at any time, with 5 replicates for the wall-time.

The same RF model as in Figure 2 was processed with endoR and `shap` using different sample sizes, $n = 500, 1000$ or 2000, and number of bootstraps for endoR, $B = 1, 10, 20, 40$ (S7C-F Fig). Furthermore, we increased the number of variables used in the predictive model by including non-selected features and fitting a new RF model via the randomForest R-package with default parameters (1) (S7A-B Fig). By default, we set $n = 1000$ and $B = 1$ bootstrap of size $n/2$ for processing with endoR, and SHAP values were calculated with the default parameters of the `shap` function (27).

Finally, the original model with 18 features and $n = 2147$ samples was processed with endoR and `shap`, with parallelization of calculations across 4 or 10 workers (controlled by the parallel R-package). For endoR, booststraps were also allowed to be run individually in parallel using the clustermq R-package (30) (option clustermq.scheduler = "multiprocessor"). Wall-times were measured from runs on a machine equipped with Intel(R) Xeon(R) E5-4620 v4 @ 2.10GHz CPUs (80 CPUs in total).

Investigation of *Methanobacteriaceae* in human gut metagenomes.

curatedMetagenome database. Data used in this chapter were downloaded from the curatedMetagenomic database (31), and all samples from Youngblut et al. (2) were included, except for samples meeting the following additional exclusion criteria: (i) samples from rectal swabs; (ii) from individuals older than 90 years old, with a BMI greater than 40 kg.m^{-2}, with any reported disease, or not part of control cohorts; (iii) samples from David et al., 2015 (32), due to the infection of all individuals with *Vibrio cholerae* or enterotoxigenic *Escherischia coli*; (iv) samples with less than a million sequence reads.

Information about sampled individuals comprised: country of origin, age, BMI, and whether the individual was from a westernized population. Here, westernization should be understood as an urban lifestyle with a diet composed of fewer carbohydrates and enriched in fat, sugar, and animal products compared to rural populations (33, 34). The dataset consisted of 2203 samples from 26 studies and 23 countries, among which 748 samples had complete gender, age, and BMI information (S2 and S3 Tables). We additionally grouped samples based on regional geographic origins, e.g. African countries grouped into Africa (S2 Table).

Enterotype clustering. Enterotypes were determined as described in Arumugam et al. (35): the Jensen–Shannon distance matrix was calculated from the relative abundances of genera using the ape (36) and phytools (37) R-packages, and partitioning around medoid was then performed with the cluster R-package (38).

Metabolic pathways formatting and filtering. Relative abundances of metabolic pathways were downloaded from the curatedMetagenomic database (31), where they had been obtained via the HUMAnN2 pipeline (39). All engineered, unmapped and unintegrated pathways were removed. Furthermore, only relative abundances of pathways at the community level, i.e., calculated from all gene abundances in the sample, were considered for analysis. Accordingly, we removed all relative abundances calculated from species-level gene abundances, i.e., the abundances attributed to distinct species (39). We additionally converted pathway abundances to 0 if their coverage was equal to 0. The HUMAnN2 pipeline calculates a confidence score that indicates whether reactions of pathways with non-zero relative abundances are confidently detected. A pathway coverage of 0 means that although genes coding for proteins involved in this pathway were detected, not all reactions of the pathway were confidently mapped (39). For this reason, for each sample and metabolic pathway, the relative abundance was replaced for 0 if the coverage was null. Finally, all pathways present in less than 25 % of samples were removed. A total of 117 metabolic pathways were included in analysis.

Taxa abundances filtering. We performed multiple taxonomic filtering steps to reduce sparsity, taxonomic redundancy, and ultimately the number of variables in the dataset.

Filtering of rare taxa We applied the same progressive filtering as aforementioned, on the pooled family, genus, and species taxonomic ranks. It allowed us to reduce the set of taxa from 3444 to 2318 (S17 Fig).

Filtering of correlated taxa from a same taxonomic branch To limit redundancy in relative abundances from taxonomic ranks of a same branch, we filtered out taxa that were significantly correlated to their direct coarser level (8). A Spearman test was performed between the two taxa, and the finer one was removed if p-value < 0.05 and $\rho^2 \geq 0.95$. A total of 89 taxa were filtered out in this manner.

***General workflow: prediction of the presence of* Methanobacteriaceae.** We looked for associations between taxa and metabolic pathways relative abundances, and metadata, with methanogen presence. Their occurrence was defined as a non-zero relative abundance of *Methanobacteriaceae*.
We fitted random forest models via the ranger R-package (40), using the case.weights parameter to account for data imbalance and with the number of trees varying in {250, 500} trees. Gradient boosted models were fitted via the XGBoost R-package (11), with the number of rounds varying in {10, 50, 100, 250, 500, 750, 1000, 1500} and the maximal depth in {1, . . . , 10}.
The performance of models were evaluated via 10 cross-validation (CV) with 70-30 % train-test splits. Model processes were fitted to training sets and predictive performance was measured using Cohen's κ on test sets (S4 Table).
For model selection, we restrained model complexity by taking into account the number of features used for fitting models and the number of trees in the forest. Decreasing the number of trees in the forest only negligibly diminished models' performance, the best model with $ntrees = 500$ had a Cohen's κ of 0.6024±0.0205 while the one with $ntrees = 250$ had a Cohen's κ of 0.6004±0.0223. The best model with $ntrees = 250$ was on average using 332.50±7.79 selected features. All next seven models were also using more than 270 features on average. However, the ninth best model in term of Cohen's κ used only 123.9±4.77 features and had a Cohen's κ very similar to the best model (Cohen's κ = 0.5957±0.0253).

Sets of predictors. We fitted models on different sets of predictors to reduce dimensionality. Since gender, age, and BMI were incomplete (S3 Table), we first assessed whether those variables were selected and used in models fitted on the 748 samples with complete information ($n_T = 500$). Otherwise, models were fitted on all samples without gender, age, and BMI. The metadata used as predictors were thus reduced to: country, region (i.e., the countries grouped by world region), westernization, enterotype (S3 Table). The original dataset name and the number of reads of each sample were included to each model processing step, even if they were not selected during FS.
To reduce noise and dimensionality, the taxa-aware feature selection step described above was performed prior to fitting predictive models.

Model interpretation with endoR. The final fitted model was processed with endoR: variables were discretized in $K = 2$ categories, bootstrapping was performed on $B = 100$ resamples, and $\alpha = 5$.

Bibliography

1. Andy Liaw, Matthew Wiener, et al. Classification and regression by randomforest. *R news*, 2(3):18–22, 2002.
2. Nicholas D Youngblut, Jacobo de la Cuesta-Zuluaga, and Ruth E Ley. Incorporating genome-based phylogeny and trait similarity into diversity assessments helps to resolve a global collection of human gut metagenomes. *bioRxiv*, 2020.
3. Pierre-Alain Chaumeil, Aaron J Mussig, Philip Hugenholtz, and Donovan H Parks. Gtdb-tk: a toolkit to classify genomes with the genome taxonomy database. 2020.
4. Jacobo de la Cuesta-Zuluaga, Ruth E Ley, and Nicholas D Youngblut. Struo: a pipeline for building custom databases for common metagenome profilers. *Bioinformatics*, 36(7):2314–2315, 2020.
5. Nan Qin, Fengling Yang, Ang Li, Edi Prifti, Yanfei Chen, Li Shao, Jing Guo, Emmanuelle Le Chatelier, Jian Yao, Lingjiao Wu, et al. Alterations of the human gut microbiome in liver cirrhosis. *Nature*, 513(7516):59–64, 2014.
6. Gabriel Al-Ghalith and Dan Knights. Burst enables optimal exhaustive dna alignment for big data. 2017. doi: doi.org/10.5281/zenodo.806850.
7. B. Jesse Shapiro, Jean Baptiste Leducq, and James Mallet. What Is Speciation? *PLoS Genetics*, 12(3):1–14, 2016. ISSN 15537404. doi: 10.1371/journal.pgen.1005860.
8. Mai Oudah and Andreas Henschel. Taxonomy-aware feature engineering for microbiome classification. *BMC bioinformatics*, 19(1):1–13, 2018.
9. Trevor Hastie, Robert Tibshirani, and Jerome Friedman. *The elements of statistical learning: data mining, inference, and prediction.* Springer Science & Business Media, New York, 2009.
10. Leo Breiman. Random forests. *Machine learning*, 45(1):5–32, 2001.

11. Tianqi Chen and Carlos Guestrin. Xgboost: A scalable tree boosting system. In *Proceedings of the 22nd acm sigkdd international conference on knowledge discovery and data mining*, pages 785–794, 2016.
12. Miron B Kursa, Witold R Rudnicki, et al. Feature selection with the boruta package. *J Stat Softw*, 36(11):1–13, 2010.
13. Houtao Deng and George Runger. Gene selection with guided regularized random forest. *Pattern Recognition*, 46(12):3483–3489, 2013.
14. Dan Knights, Elizabeth K Costello, and Rob Knight. Supervised classification of human microbiota. *FEMS microbiology reviews*, 35(2):343–359, 2011.
15. Davide Albanese, Carlotta De Filippo, Duccio Cavalieri, and Claudio Donati. Explaining diversity in metagenomic datasets by phylogenetic-based feature weighting. *PLoS Comput Biol*, 11(3): e1004186, 2015.
16. Thomas Hooven, Yun Chao Lin, and Ansaf Salleb-Aouissi. Multiple instance learning for predicting necrotizing enterocolitis in premature infants using microbiome data. In *Proceedings of the ACM Conference on Health, Inference, and Learning*, pages 99–109, 2020.
17. Jakob Wirbel, Konrad Zych, Morgan Essex, Nicolai Karcher, Ece Kartal, Guillem Salazar, Peer Bork, Shinichi Sunagawa, and Georg Zeller. Microbiome meta-analysis and cross-disease comparison enabled by the siamcat machine learning toolbox. *Genome biology*, 22(1):1–27, 2021.
18. Robert Tibshirani. Regression shrinkage and selection via the lasso. *Journal of the Royal Statistical Society: Series B (Methodological)*, 58(1):267–288, 1996.
19. Jerome Friedman, Trevor Hastie, and Rob Tibshirani. Regularization paths for generalized linear models via coordinate descent. *Journal of statistical software*, 33(1):1, 2010.
20. Jerome Friedman, Trevor Hastie, and Robert Tibshirani. Sparse inverse covariance estimation with the graphical lasso. *Biostatistics*, 9(3):432–441, 2008.
21. Jonathan Friedman and Eric J Alm. Inferring correlation networks from genomic survey data. *PLoS Comput Biol*, 8(9):e1002687, 2012.
22. Zachary D Kurtz, Christian L Müller, Emily R Miraldi, Dan R Littman, Martin J Blaser, and Richard A Bonneau. Sparse and compositionally robust inference of microbial ecological networks. *PLoS Comput Biol*, 11(5):e1004226, 2015.
23. Nicolai Meinshausen, Peter Bühlmann, et al. High-dimensional graphs and variable selection with the lasso. *Annals of statistics*, 34(3):1436–1462, 2006.
24. Leo Breiman and Adele Cutler. Manual on setting up, using, and understanding random forests, ver. 4.0, 2003.
25. Scott M Lundberg, Bala Nair, Monica S Vavilala, Mayumi Horibe, Michael J Eisses, Trevor Adams, David E Liston, Daniel King-Wai Low, Shu-Fang Newman, Jerry Kim, and Su-In Lee. Explainable machine-learning predictions for the prevention of hypoxaemia during surgery. *Nat Biomed Eng*, 2(10):749–760, October 2018.
26. Scott M Lundberg, Gabriel Erion, Hugh Chen, Alex DeGrave, Jordan M Prutkin, Bala Nair, Ronit Katz, Jonathan Himmelfarb, Nisha Bansal, and Su-In Lee. From local explanations to global understanding with explainable AI for trees. *Nature Machine Intelligence*, 2(1):56–67, January 2020.
27. Przemyslaw Biecek and Tomasz Burzykowski. Explanatory model analysis. https://pbiecek.github.io/ema/preface.html, March 2020. Accessed: 2020-4-28.
28. Christoph Molnar, Giuseppe Casalicchio, and Bernd Bischl. iml: An r package for interpretable machine learning. *Journal of Open Source Software*, 3(26):786, 2018.
29. Yang Liu and Allan Just. *SHAPforxgboost: SHAP Plots for 'XGBoost'*, 2020. R package version 0.1.0.
30. Michael Schubert. clustermq: Evaluate Function Calls on HPC Schedulers (LSF, SGE, SLURM, PBS/Torque), 2020.
31. Edoardo Pasolli, Lucas Schiffer, Paolo Manghi, Audrey Renson, Valerie Obenchain, Duy Tin Truong, Francesco Beghini, Faizan Malik, Marcel Ramos, Jennifer B Dowd, et al. Accessible, curated metagenomic data through experimenthub. *Nature methods*, 14(11):1023, 2017.
32. Lawrence A David, Ana Weil, Edward T Ryan, Stephen B Calderwood, Jason B Harris, Fahima Chowdhury, Yasmin Begum, Firdausi Qadri, Regina C LaRocque, and Peter J Turnbaugh. Gut microbial succession follows acute secretory diarrhea in humans. *mBio*, 6(3):00381–15, 5 2015. ISSN 2150-7511. doi: 10.1128/mBio.00381-15.
33. Barry M Popkin. The nutrition transition and its health implications in lower-income countries. *Public Health Nutrition*, 1(1):5–21, 1998. ISSN 1368-9800. doi: 10.1079/phn19980004.
34. Edoardo Pasolli, Francesco Asnicar, Serena Manara, Moreno Zolfo, Nicolai Karcher, Federica Armanini, Francesco Beghini, Paolo Manghi, Adrian Tett, Paolo Ghensi, et al. Extensive unexplored human microbiome diversity revealed by over 150,000 genomes from metagenomes spanning age, geography, and lifestyle. *Cell*, 176(3):649–662, 2019.
35. Manimozhiyan Arumugam, Jeroen Raes, Eric Pelletier, Denis Le Paslier, Takuji Yamada, Daniel R Mende, Gabriel R Fernandes, Julien Tap, Thomas Bruls, Jean-Michel Batto, Marcelo Bertalan, Natalia Borruel, Francesc Casellas, Leyden Fernandez, Laurent Gautier, Torben Hansen, Masahira Hattori, Tetsuya Hayashi, Michiel Kleerebezem, Ken Kurokawa, Marion Leclerc, Florence Levenez, Chaysavanh Manichanh, H. Bjørn Nielsen, Trine Nielsen, Nicolas Pons, Julie Poulain, Junjie Qin, Thomas Sicheritz-Ponten, Sebastian Tims, David Torrents, Edgardo Ugarte, Erwin G Zoetendal, Jun Wang, Francisco Guarner, Oluf Pedersen, Willem M. de Vos, Søren Brunak, Joel Doré, Jean Weissenbach, S Dusko Ehrlich, and Peer Bork. Enterotypes of the human gut microbiome. *Nature*, 473(7346):174–180, 5 2011. ISSN 0028-0836. doi: 10.1038/nature09944.
36. Emmanuel Paradis and Klaus Schliep. ape 5.0: an environment for modern phylogenetics and evolutionary analyses in R. *Bioinformatics*, 35(3):526–528, 2 2019. ISSN 1367-4803. doi: 10.1093/bioinformatics/bty633.
37. Liam J. Revell. phytools: an R package for phylogenetic comparative biology (and other things). *Methods in Ecology and Evolution*, 3(2):217–223, 4 2012. ISSN 2041210X. doi: 10.1111/j.2041-210X.2011.00169.x.
38. M. Maechler, P. Rousseeuw, A. Struyf, M. Hubert, and K. Hornik. cluster: Cluster Analysis Basics and Extensions, 2019.
39. Eric A Franzosa, Lauren J McIver, Gholamali Rahnavard, Luke R Thompson, Melanie Schirmer, George Weingart, Karen Schwarzberg Lipson, Rob Knight, J Gregory Caporaso, Nicola Segata, et al. Species-level functional profiling of metagenomes and metatranscriptomes. *Nature methods*, 15(11):962–968, 2018.
40. Marvin N. Wright and Andreas Ziegler. ranger : A Fast Implementation of Random Forests for High Dimensional Data in C++ and R. *Journal of Statistical Software*, 77(1), 2017. ISSN 1548-7660. doi: 10.18637/jss.v077.i01.
