# Supplementary material for: Interpreting tree ensemble machine learning models with endoR
Source: PLoS Comput Biol. 2022 Dec 14;18(12):e1010714. doi: 10.1371/journal.pcbi.1010714 (PMC9797088; doi:10.1371/journal.pcbi.1010714)
Supplement: S3 Text — (PDF) [file pcbi.1010714.s003.pdf]

Interpreting tree ensemble machine learning models with endoR - S3 Text

Albane Ruaud[a], Niklas Pfister[b], Ruth E Ley[a], Nicholas D Youngblut[a,*]

[a]Max Planck Institute for Developmental Biology, Department of Microbiome Science, Tuebingen, Germany
[b]University of Copenhagen, Department of Mathematical Sciences, Copenhagen, Denmark
* nicholas.youngblut@tuebingen.mpg.de

Supplementary Results

Evaluation of endoR.

Higher number of boostraps reduce overfitting. We generated 100 FSDs and 50 APs datasets and processed them with endoR with $B = 10$, 50 or 100 for FSDs (Fig 3D) and with $B = 10$ or $B = 100$ for APs (S5F Fig). Varying the number of bootstrap resamples between 10 and 90 did not affect the precision and recall of endoR (S5F Fig), although higher bootstrap numbers decreased the overfitting of results (S6 Fig). This consistency in results suggests that (i) on average, endoR results are similar for different number of bootstraps, and (ii) our stability selection procedure is efficient at discriminating relevant decisions. However, increasing the number of bootstraps aids with obtaining steady decision ensembles. This slight decrease in variance, given higher number of bootstraps, is exemplified in a subsequent analysis, where we repeatedly processed replicates of the artificial phenotypes using distinct bootstrap resamples, for $B = 10$ or 100 bootstraps each time (S6 Fig). Therefore, although endoR outputs similar results regardless of the number of bootstraps, those results are more likely to be closer to the expected average results with higher number of bootstraps.

Discretization only marginally affects endoR. Finally, we assessed the effect of discretization. This optional step eases the interpretation of endoR outputs by simplifying continuous variables into categorical ones (e.g., ‘Low’ versus ‘High’ values grouped together). However, as this operation results in numeric variables being replaced by categories in decisions, the support and prediction of decisions are also affected. Consequently, all downstream endoR regularization steps may generate alternative stable decision ensembles for different discretization procedures. We processed the 50 APs and 100 FSDs with endoR using a discretization in K = 2 or 3 categories based on the distribution of each numeric variable. Our simulations showed that the precision of endoR results were similar (S5H and J Fig). Nonetheless, the recall was slightly higher when discretization was performed in three categories (S5G and I Fig), likely due to the thinner mapping of categorical variables to their original numeric ones with more categories. Increasing the number of categories also means that higher computation resources are needed: each decision may be multiplied by a factor 1 for $K = 2$ but by a factor up to 2^{p_D} for $K = 3$, p_D the number of variables in the decision D. We note that several methods exist to discretize data. While here, and in the rest of the article, we used the quantiles of variables’ original distribution to discretize data, we propose an alternative method in the S1 Text, that we compare to the present one in the next paragraph and S5G-J Fig.
Alternatively, numeric variables can be discretized using the input predictive model. For each variable, the thresholds used in the model to make splits are employed to define the new variable categories (see S1 Text). For neither of the FSD and AP datasets did the method affect endoR results (S5G-J Fig). Both methods are available in our package under the parameter ‘mode’, with discretization based on data distribution as default.

New insights into the ecology of human gut methanogens.

Individuals weakly cluster into enterotypes. We explored the spread of samples along the enterotype landscape (1, 2). Similar to previous findings (1, 2), the Jensen–Shannon distance calculated from the relative abundances of genera separated observations according to gradients of enrichment in *Bacteroides* and *Prevotella* (S18A-B Fig). However, samples did not strongly cluster, as shown by the within-group silhouette scores below 0.5, indicating weak clustering (2, 3) (S18D-G Fig). This was to be expected due to the heterogeneity of studies included in the meta-analysis and is consistent with the low silhouette scores reported for these same data (4). Clustering in three groups resulted in sample groups consistent with the ETB, ETF, and ETP enterotypes previously reported as mapping onto the gradients in *Bacteroides* and *Prevotella* relative abundances (1, 2) (S18A-C Fig). Since the ETF enterotype has been positively associated with higher relative abundances of *M. smithii* (2), despite the enterotypes low homogeneity, they were included in further analysis to verify their association with the methanogen.

Bibliography

1. Manimozhiyan Arumugam, Jeroen Raes, Eric Pelletier, Denis Le Paslier, Takuji Yamada, Daniel R Mende, Gabriel R Fernandes, Julien Tap, Thomas Bruls, Jean-Michel Batto, Marcelo Bertalan, Natalia Borruel, Francesc Casellas, Leyden Fernandez, Laurent Gautier, Torben Hansen, Masahira Hattori, Tetsuya Hayashi, Michiel Kleerebezem, Ken Kurokawa, Marion Leclerc, Florence Levenez, Chaysavanh Manichanh, H. Bjørn Nielsen, Trine Nielsen, Nicolas Pons, Julie Poulain, Junjie Qin, Thomas Sicheritz-Ponten, Sebastian Tims, David Torrents, Edgardo Ugarte, Erwin G Zoetendal, Jun Wang, Francisco Guarner, Oluf Pedersen, Willem M. de Vos, Søren Brunak, Joel Doré, Jean Weissenbach, S Dusko Ehrlich, and Peer Bork. Enterotypes of the human gut microbiome. *Nature*, 473 (7346):174–180, 5 2011. ISSN 0028-0836. doi: 10.1038/nature09944.

2. Paul I Costea, Falk Hildebrand, Manimozhiyan Arumugam, Fredrik Bäckhed, Martin J Blaser, Frederic D Bushman, Willem M De Vos, S Dusko Ehrlich, Claire M Fraser, Masahira Hattori, et al. Enterotypes in the landscape of gut microbial community composition. *Nature microbiology*, 3(1):8–16, 2018.
3. Omry Koren, Dan Knights, Antonio Gonzalez, Levi Waldron, Nicola Segata, Rob Knight, Curtis Huttenhower, and Ruth E. Ley. A Guide to Enterotypes across the Human Body: Meta-Analysis of Microbial Community Structures in Human Microbiome Datasets. *PLoS Computational Biology*, 9(1):e1002863, 1 2013. ISSN 1553-7358. doi: 10.1371/journal.pcbi.1002863.
4. Edoardo Pasolli, Lucas Schiffer, Paolo Manghi, Audrey Renson, Valerie Obenchain, Duy Tin Truong, Francesco Beghini, Faizan Malik, Marcel Ramos, Jennifer B Dowd, et al. Accessible, curated metagenomic data through experimenthub. *Nature methods*, 14(11):1023, 2017.
