# Supplementary material for: Interpreting tree ensemble machine learning models with endoR
Source: PLoS Comput Biol. 2022 Dec 14;18(12):e1010714. doi: 10.1371/journal.pcbi.1010714 (PMC9797088; doi:10.1371/journal.pcbi.1010714)
Supplement: S4 Text — (PDF) [file pcbi.1010714.s004.pdf]

Interpreting tree ensemble machine learning models with endoR - S4 Text

Albane Ruaud[a], Niklas Pfister[b], Ruth E Ley[a], Nicholas D Youngblut[a,*]

[a]Max Planck Institute for Developmental Biology, Department of Microbiome Science, Tuebingen, Germany
[b]University of Copenhagen, Department of Mathematical Sciences, Copenhagen, Denmark
* nicholas.youngblut@tuebingen.mpg.de

Abbreviations

- AP: artificial phenotype
- BMI: body mass index
- CV: cross-validation
- DNA: deoxyribonucleic acid
- FN: false negative
- FP: false positive
- FS: feature selection
- FSD: fully simulated dataset
- ML: machine Learning
- RF: random forest
- TN: true negative
- TP: true positive
