# Supplementary material for: Interpreting tree ensemble machine learning models with endoR
Source: PLoS Comput Biol. 2022 Dec 14;18(12):e1010714. doi: 10.1371/journal.pcbi.1010714 (PMC9797088; doi:10.1371/journal.pcbi.1010714)
Supplement: S1 Fig — A/ Ten sets of observations, each containing a subset for training and one for testing, were created. Training observations were used to fit models, i.e., the combination of a feature selection and classifier algorithms for given hyperparameter values, and testing ones were predicted with the fitted models. Model’s performances were averaged across testing sets. Feature selection algorithms consisted of (i) no feature selection, (ii) a taxa-aware version of the gRRF algorithm [21] (S2 Text), (iii) the Boruta algorithm [24], and (iv) no feature selection. Classifiers were fitted with random forests or gradient boosted model algorithms. Metadata correspond to the number of reads and original dataset names. B/ The model (feature selection algorithm and classifier) that resulted in A/ in the highest average Cohen’s κ using the fewest features was used to fit the final classifier on all data. (PDF) [file pcbi.1010714.s005.pdf]

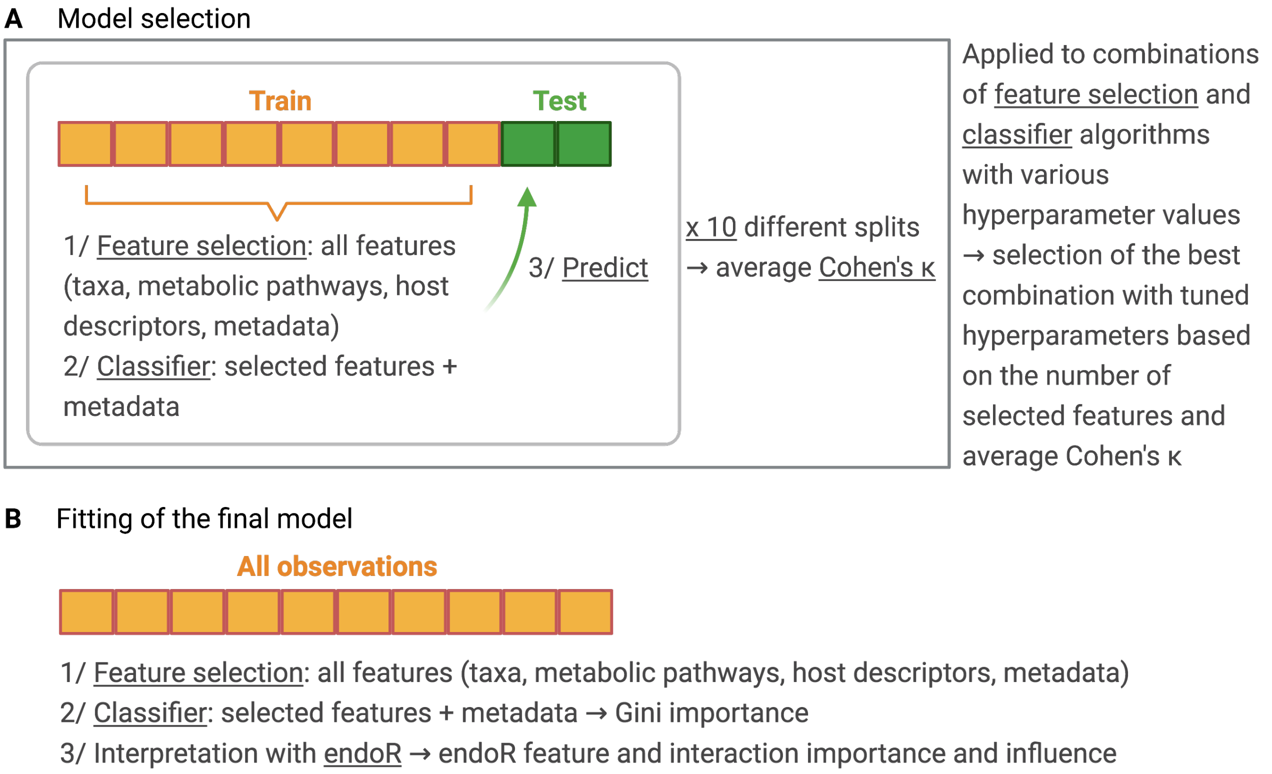

Figure S1. Model selection and fitting for predicting the presence/absence of *Methanobacteriaceae*. A/ Ten sets of observations, each containing a subset for training and one for testing, were created. Training observations were used to fit models, i.e., the combination of a feature selection and classifier algorithms for given hyperparameter values, and testing ones were predicted with the fitted models. Model's performances were averaged across testing sets. Feature selection algorithms consisted of (i) no feature selection, (ii) a taxa-aware version of the gRRF algorithm (1) (S2 Text), (iii) the Boruta algorithm (2), (iv) no feature selection. Classifiers were fitted with random forests or gradient boosted model algorithms. Metadata correspond to the number of reads and original dataset names. B/ The model (feature selection algorithm and classifier) that resulted in A/ in the highest average Cohen's κ using the fewest features was used to fit the final classifier on all data.

References

1. Houtao Deng and George Runger. Gene selection with guided regularized random forest. *Pattern Recognition*, 46(12):3483–3489, 2013.
2. Miron B Kursa, Witold R Rudnicki, et al. Feature selection with the boruta package. *J Stat Softw*, 36(11):1–13, 2010.
