# Supplementary material for: Interpreting tree ensemble machine learning models with endoR
Source: PLoS Comput Biol. 2022 Dec 14;18(12):e1010714. doi: 10.1371/journal.pcbi.1010714 (PMC9797088; doi:10.1371/journal.pcbi.1010714)
Supplement: S2 Fig — endoR was applied to the set of rules directly obtained from the true mechanism generating the response variables for one replicate of each of the AP (A-B) and FSD (C-D) simulations. No regularization step was performed, i.e., no pruning nor bootstrapping. Respective ground truth networks are visualised in Fig 2F and S4F Fig. The additional edges on B are due to the discretization step. No additional edge appears on D due to the proximity between the median of numeric features (used to discretize data) and the thresholds used to make the response variable. (PDF) [file pcbi.1010714.s006.pdf]

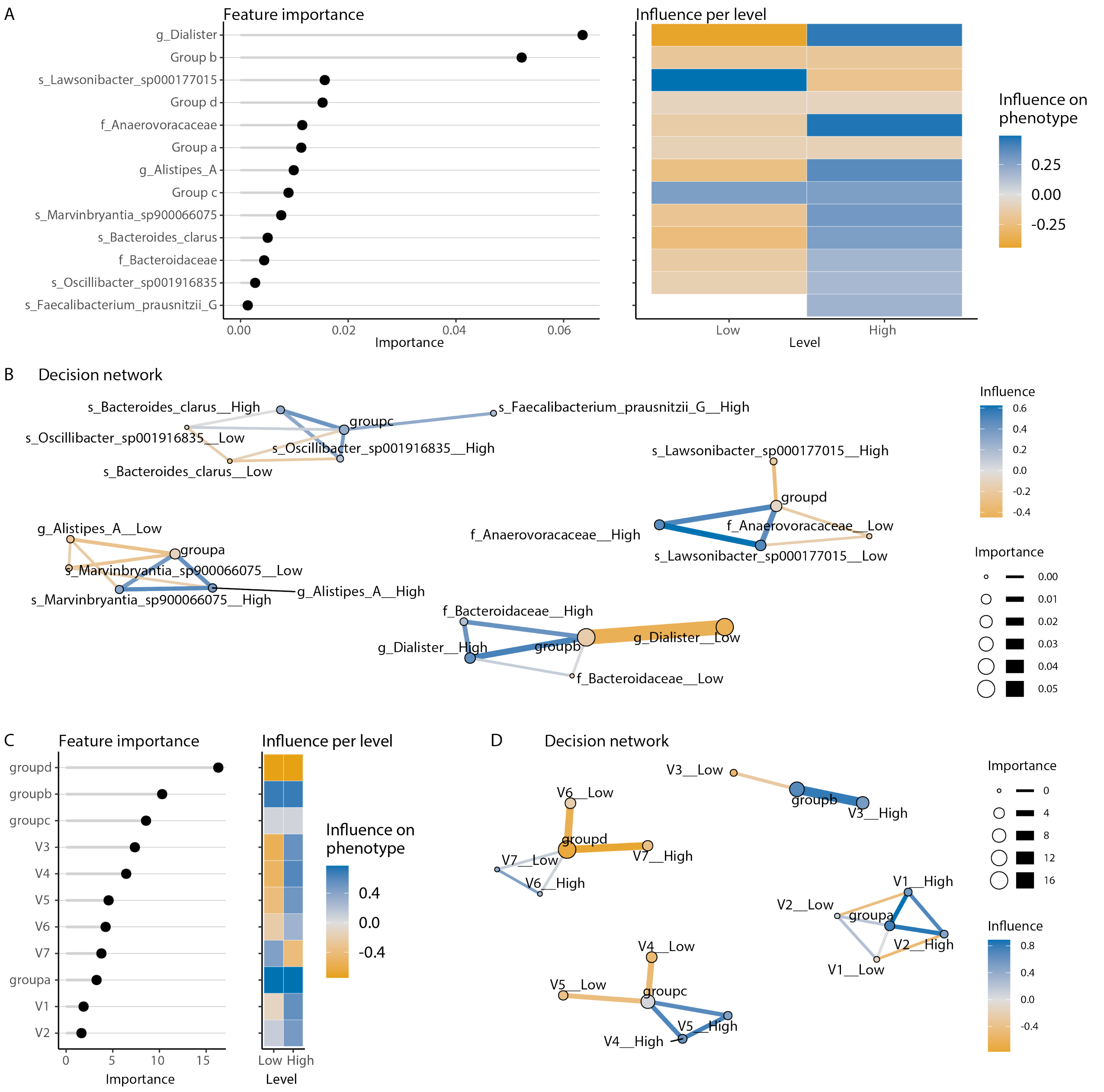

Figure S2. endoR recovers ground truth network from perfect predictive models. endoR was applied to the set of rules directly obtained from the true mechanism generating the response variables for one replicate of each of the AP (A-B) and FSD (C-D) simulations. No regularization step was performed, i.e., no pruning nor bootstrapping. Respective ground truth networks are visualised in Figs 2F and S4F. The additional edges on B are due to the discretization step. No additional edge appears on D due to the proximity between the median of numeric features (used to discretize data) and the thresholds used to make the response variable.
