# Supplementary material for: Interpreting tree ensemble machine learning models with endoR
Source: PLoS Comput Biol. 2022 Dec 14;18(12):e1010714. doi: 10.1371/journal.pcbi.1010714 (PMC9797088; doi:10.1371/journal.pcbi.1010714)
Supplement: S3 Fig — Global null models were generated from 10 APs by randomizing the target values within each group (see Methods). A predictive model was then fitted, including a FS step followed by the fitting of a RF classifier. Models were interpreted with endoR: no stable decision ensemble was reached in 6/10 cases, a unique stable decision was found in 3/10 cases, and a stable decision ensemble was found for the replicate which had the highest RF accuracy. (PDF) [file pcbi.1010714.s007.pdf]

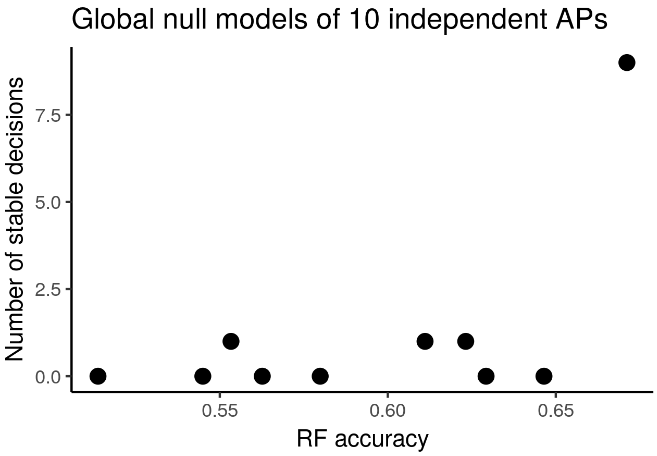

Figure S3. endoR generally does not find stable decision ensemble from global null models. Global null models were generated from 10 APs by randomizing the target values within each group (see Methods). A predictive model was then fitted, including a FS step followed by the fitting of a RF classifier. Models were interpreted with endoR: no stable decision ensemble was reached in 6/10 cases, a unique stable decision was found in 3/10 cases, and a stable decision ensemble was found for the replicate which had the highest RF accuracy.
