# Supplementary material for: Interpreting tree ensemble machine learning models with endoR
Source: PLoS Comput Biol. 2022 Dec 14;18(12):e1010714. doi: 10.1371/journal.pcbi.1010714 (PMC9797088; doi:10.1371/journal.pcbi.1010714)
Supplement: S4 Fig — A-D/ Fully simulated data (FSD) structure: four groups of samples (labelled from a to d) were generated so that for each group, the binary response variable takes the value ‘1’ (blue) or ‘-1’ (yellow) according to a combination of variables described in Table 1 (e.g., V1 and V2 for Group a). The values of the response variable were then randomized with a probability r = 0.05. E/ Ground truth network of associations between the response variable and single variables (nodes) and pairs of variables (edges) described in A/ (see Methods). Pairs of variables predicting ‘1’ are linked by a blue edge (‘positive’) and those predicting ‘-1’ by a yellow edge (‘negative’). Variables for which high values are predictive of ‘1’ have a blue node color (‘positive’) and a yellow node color if high values are predictive of ‘-1’ (‘negative’). If high values are predictive of ‘1’ or ‘-1’ depending of other variable values (e.g., Group b predicts ‘1’ if V3 takes high values, but ‘-1’ if V3 has low values), the color is grey (‘depends’). F/ Feature importance as measured by the mean decrease in Gini impurity in the fitted random forest (RF) model trained on the dataset shown in A/. G/ Feature importance as measured by endoR and feature influences for each discretized level of numeric variables as computed by endoR. The point color indicates whether features were used to construct the response (‘True’) or not (‘Irrelevant’). H/ Decision network produced by endoR. Edges and nodes correspond to single variables and their interaction effect on the response variable, respectively. Edge widths and node sizes are proportional to the interaction and feature importances calculated by endoR, respectively; their colors are representative of their influence (see Methods in main text and S2 Text for details on network construction). The edge transparency is inversely proportional to the importance for H only. I/ Same than H but edges with lowest interaction importance were removed to obtain paths bet [file pcbi.1010714.s008.pdf]

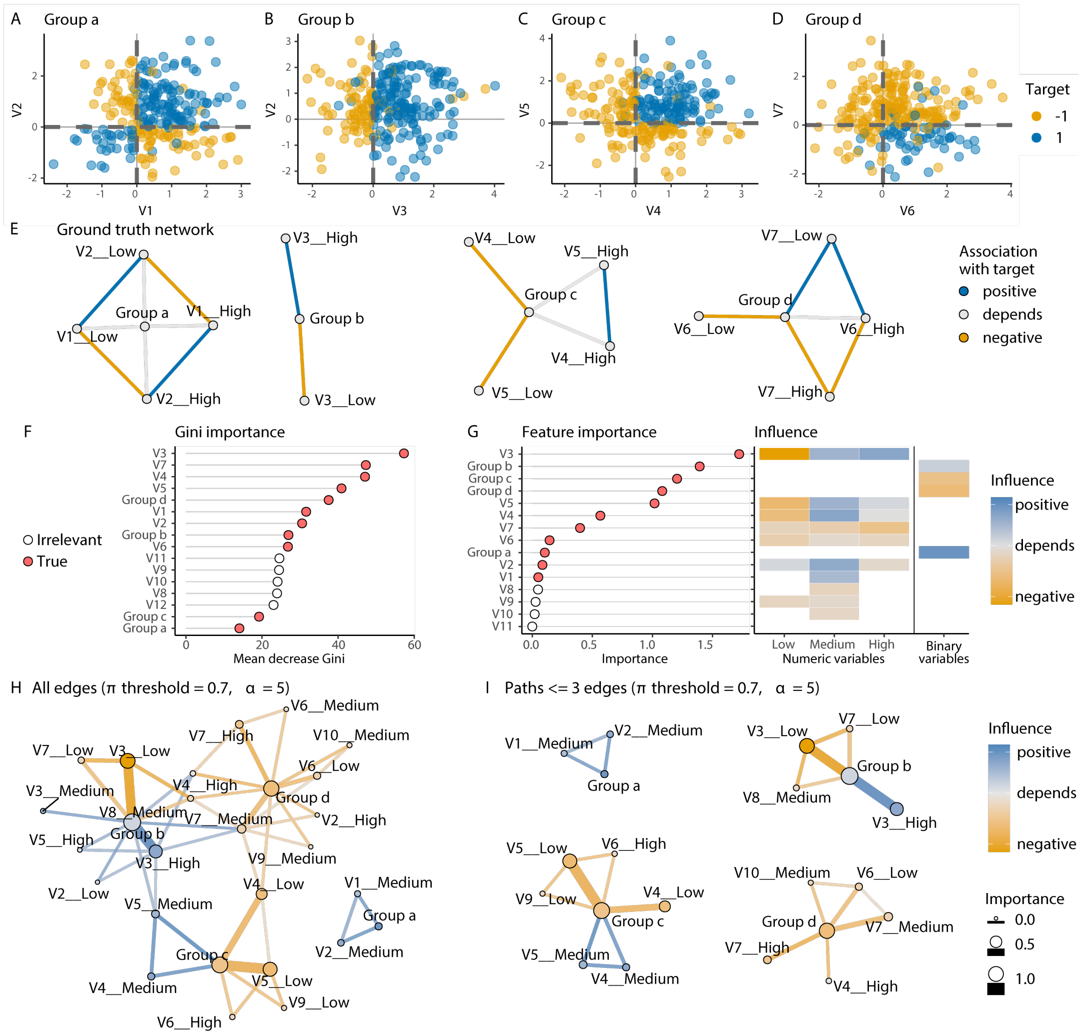

Figure S4. endoR captures interactions predictive of a response variable from a random forest fitted on simulated data. A-D/ Fully simulated data (FSD) structure: four groups of samples (labelled from a to d) were generated so that for each group, the binary response variable takes the value '1' (blue) or '-1' (yellow) according to a combination of variables described in Table 1 (e.g., V1 and V2 for Group a). The values of the response variable were then randomized with a probability $r = 0.05$. E/ Ground truth network of associations between the response variable and single variables (nodes) and pairs of variables (edges) described in A/ (see Methods). Pairs of variables predicting '1' are linked by a blue edge ('positive') and those predicting '-1' by a yellow edge ('negative'). Variables for which high values are predictive of '1' have a blue node color ('positive') and a yellow node color if high values are predictive of '-1' ('negative'). If high values are predictive of '1' or '-1' depending of other variable values (e.g., Group b predicts '1' if V3 takes high values, but '-1' if V3 has low values), the color is grey ('depends'). F/ Feature importance as measured by the mean decrease in Gini impurity in the fitted random forest (RF) model trained on the dataset shown in A/. G/ Feature importance as measured by endoR and feature influences for each discretized level of numeric variables as computed by endoR. The point color indicates whether features were used to construct the response ('True') or not ('Irrelevant'). H/ Decision network produced by endoR. Edges and nodes correspond to single variables and their interaction effect on the response variable, respectively. Edge widths and node sizes are proportional to the interaction and feature importances calculated by endoR, respectively; their colors are representative of their influence (see Methods in main text and S2 Text for details on network construction). The edge transparency is inversely proportional to the importance for H only. I/ Same than H but edges with lowest interaction importance were removed to obtain paths between nodes of length ≤ 3. E, G-I/ Levels of discretized variables, i.e., numeric variables transformed into categorical variables based on their quantiles, are shown on the x-axis of the influence plot (G/) and indicated by '__High' or '__Low' in networks (E/ and H/).
