# Supplementary material for: Interpreting tree ensemble machine learning models with endoR
Source: PLoS Comput Biol. 2022 Dec 14;18(12):e1010714. doi: 10.1371/journal.pcbi.1010714 (PMC9797088; doi:10.1371/journal.pcbi.1010714)
Supplement: S5 Fig — 100 FSDs (B-E and G-H/) and 50 APs (A, F, and I-J/) were generated, RFs were fitted and processed with endoR. If not varied, parameters were as follow: ntree = 500, discretization was performed with the method based on the data distribution with K = 2 categories, and α = 5. We computed the following three metrics: Cohen’s κ of the RF, weighted precision and recall values of the selected edges in the stable decision ensemble, and TP/FP-curves based on the probabilities of being selected in the stable decision ensemble (see Methods). A-B, D-E, and G-J/ TP/FP-curves are averaged across all datasets for a fixed parameter setting (line) and standard deviation (shaded area) are displayed. The average number of TPs and FPs expected for a randomization null model and standard deviations, are shown in grey. Large points indicate the average number of TPs and FPs in the stable ensembles generated by endoR. C and F/ Each point corresponds to the precision/recall of endoR applied to a single dataset and parameter setting. The larger traced points are the averages across all datasets for a fixed parameter setting. A-B and D-E/ As expected decreasing the noise or increasing the number of trees in the forest improves the performance of endoR both in terms of precision and recall. Importantly, there is a strong dependence of endoR performance on the performance of the fitted RF and endoR. Moreover, endoR has a good precision even for small RF. C/ Increasing α increases both the TPs and FPs. Small values of α effectively control the FPs without strongly impacting the recovered TPs. F/ Larger values of B are slightly better but endoR performs well even for small values of B. G-J/ Discretization was performed by creating K = 2 or 3 categories from numeric variables based on their distribution (‘data’) or on the splits on these variables in the fitted RF (‘RF thr’). Discretization slightly influences endoR performance, without any clear pattern between the FSD and AP simulations. (PDF) [file pcbi.1010714.s009.pdf]

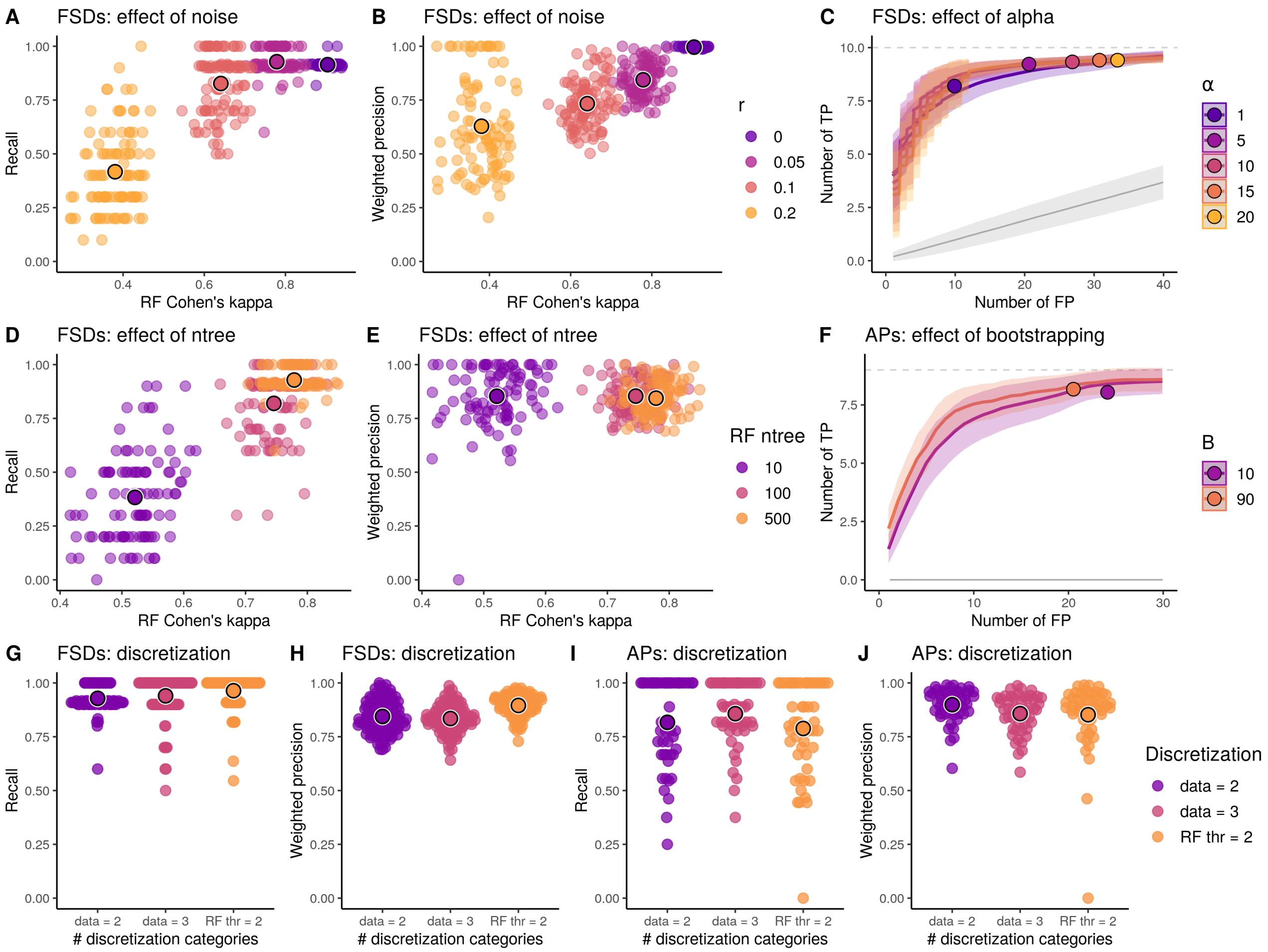

Figure S5. The accuracy of endoR increases with the accuracy of input models. 100 FSDs (B-E and G-H/) and 50 APs (A, F, and I-J/) were generated, RFs were fitted and processed with endoR. If not varied, parameters were as follow: $ntree$ = 500, discretization was performed with the method based on the data distribution with $K = 2$ categories, and $\alpha = 5$. We computed the following three metrics: Cohen's κ of the RF, weighted precision and recall values of the selected edges in the stable decision ensemble, and TP/FP-curves based on the probabilities of being selected in the stable decision ensemble (see Methods). A-B, D-E, and G-J/ TP/FP-curves are averaged across all datasets for a fixed parameter setting (line) and standard deviation (shaded area) are displayed. The average number of TPs and FPs expected for a randomization null model and standard deviations, are shown in grey. Large points indicate the average number of TPs and FPs in the stable ensembles generated by endoR. C and F/ Each point corresponds to the precision/recall of endoR applied to a single dataset and parameter setting. The larger traced points are the averages across all datasets for a fixed parameter setting. A-B and D-E/ As expected decreasing the noise or increasing the number of trees in the forest improves the performance of endoR both in terms of precision and recall. Importantly, there is a strong dependence of endoR performance on the performance of the fitted RF and endoR. Moreover, endoR has a good precision even for small RF. C/ Increasing α increases both the TPs and FPs. Small values of α effectively control the FPs without strongly impacting the recovered TPs. F/ Larger values of B are slightly better but endoR performs well even for small values of B. G-J/ Discretization was performed by creating K = 2 or 3 categories from numeric variables based on their distribution ('data') or on the splits on these variables in the fitted RF ('RF thr'). Discretization slightly influences endoR performance, without any clear pattern between the FSD and AP simulations.
