# Supplementary material for: Interpreting tree ensemble machine learning models with endoR
Source: PLoS Comput Biol. 2022 Dec 14;18(12):e1010714. doi: 10.1371/journal.pcbi.1010714 (PMC9797088; doi:10.1371/journal.pcbi.1010714)
Supplement: S6 Fig — A total of 6 replicates of artificial phenotypes were each processed 10 times with B = 10 or 100 bootstraps resamples (purple and orange, respectively). The curves show the average number (‘#’) of identified true positive (TP) and false positive (FP) edges according to edge probabilities of being selected in the stable decision ensemble. Curves were interpolated for each technical replicate, and the average (line) and standard deviation (shaded area) across number of bootstraps are displayed. The traced points denote average number of TP and FP in the stable ensembles returned by endoR for π = 0.7 and α = 5. (PDF) [file pcbi.1010714.s010.pdf]

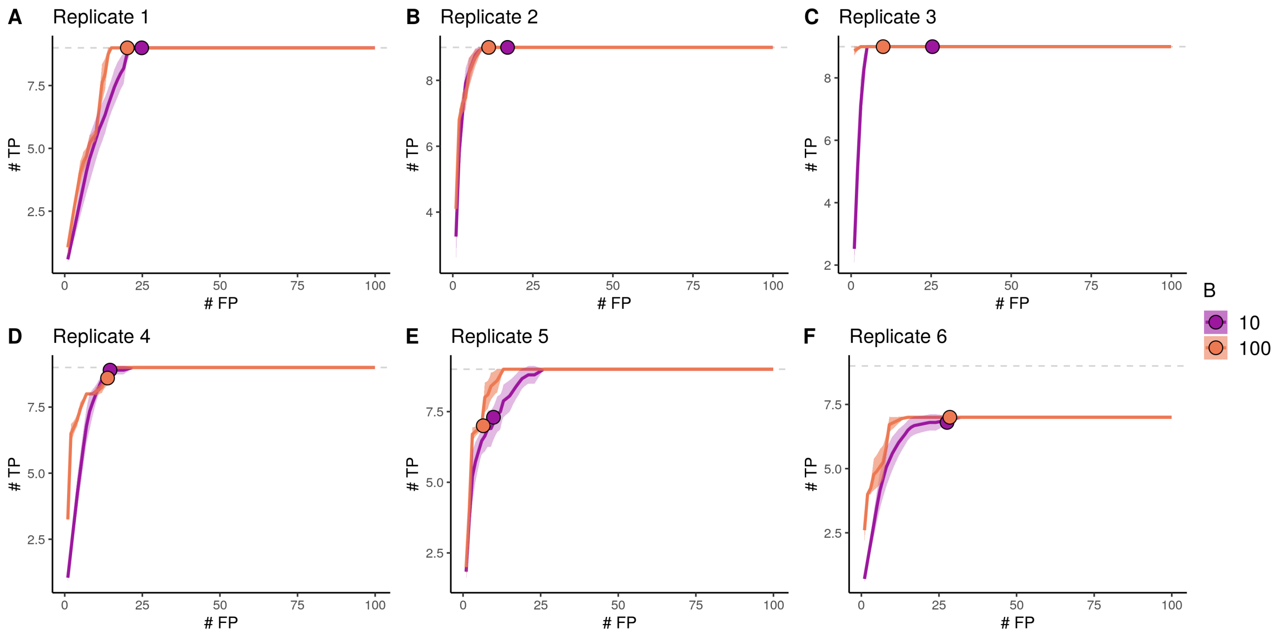

Figure S6. endoR performance stabilizes as the number of bootstraps increases. A total of 6 replicates of artificial phenotypes were each processed 10 times with $B = 10$ or 100 bootstraps resamples (purple and orange, respectively). The curves show the average number ('#') of identified true positive (TP) and false positive (FP) edges according to edge probabilities of being selected in the stable decision ensemble. Curves were interpolated for each technical replicate, and the average (line) and standard deviation (shaded area) across number of bootstraps are displayed. The traced points denote average number of TP and FP in the stable ensembles returned by endoR for $\pi = 0.7$ and $\alpha = 5$.
