# Supplementary material for: Interpreting tree ensemble machine learning models with endoR
Source: PLoS Comput Biol. 2022 Dec 14;18(12):e1010714. doi: 10.1371/journal.pcbi.1010714 (PMC9797088; doi:10.1371/journal.pcbi.1010714)
Supplement: S7 Fig — A-F/ The total CPU time and maximal virtual memory used for three replicate processing runs of the same RF model. The artificial phenotype presented in Fig 2 was used with 18 variables and 1000 samples (see Methods), and endoR was run on B = 1 bootstrap of size n/2. G/ Five technical replicates of endoR and shap runs on the RF trained to predict the artificial phenotype presented in Fig 2 (18 variables and 2147 observations). Calculations were ran in parallel across 4 or 10 workers; for endoR, bootstraps were further ran individually in parallel (see S2 Text). (PDF) [file pcbi.1010714.s011.pdf]

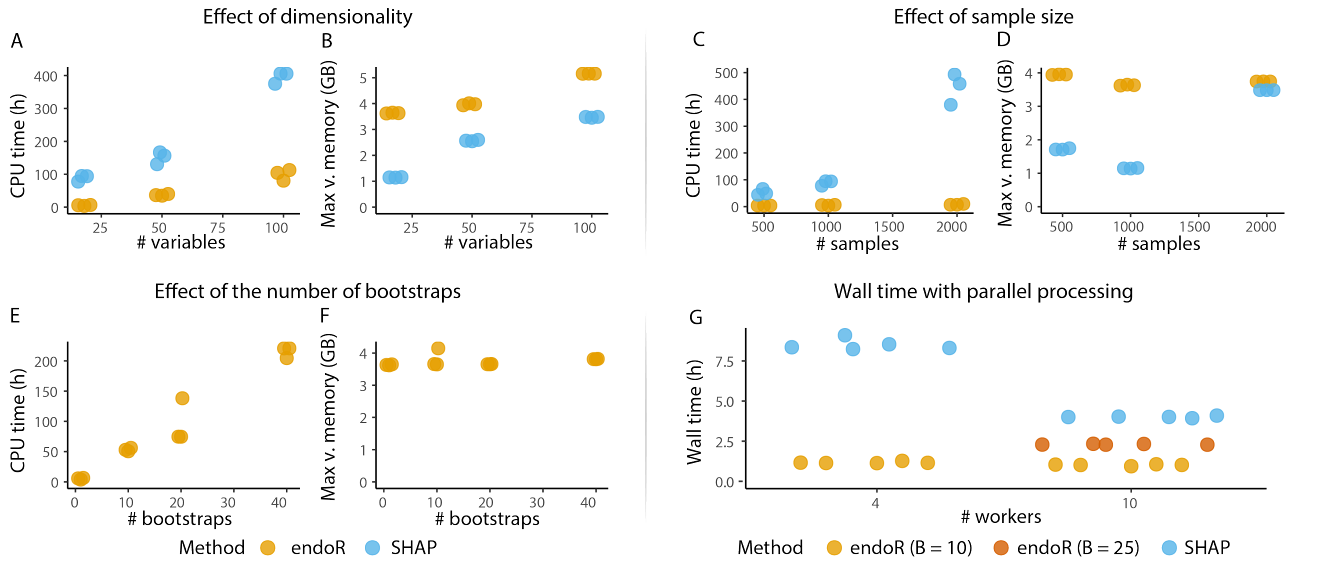

Figure S7. endoR computation time scales substantially better than SHAP when applied to random forest classifiers. A-F/ The total CPU time and maximal virtual memory used for three replicate processing runs of the same RF model. The artificial phenotype presented in Fig 2 was used with 18 variables and 1000 samples (see Methods), and endoR was run on $B = 1$ bootstrap of size $n/2$. G/ Five technical replicates of endoR and `shap` runs on the RF trained to predict the artificial phenotype presented in Fig 2 (18 variables and 2147 observations). Calculations were ran in parallel across 4 or 10 workers; for endoR, bootstraps were further ran individually in parallel (see S2 Text).
