# Supplementary material for: Interpreting tree ensemble machine learning models with endoR
Source: PLoS Comput Biol. 2022 Dec 14;18(12):e1010714. doi: 10.1371/journal.pcbi.1010714 (PMC9797088; doi:10.1371/journal.pcbi.1010714)
Supplement: S8 Fig — Simple example of the discretization of a uniformly distributed variable x into three levels. An original rule “x < t” (orange) is modified according to the number of observations in each level included in the sample support of the rule (new rule-s in green). B/ A minority of samples in the “Medium” level were included in the original sample support defined by “x < t”, therefore the “Medium” level is not selected to make a new rule as in C/. (PDF) [file pcbi.1010714.s012.pdf]

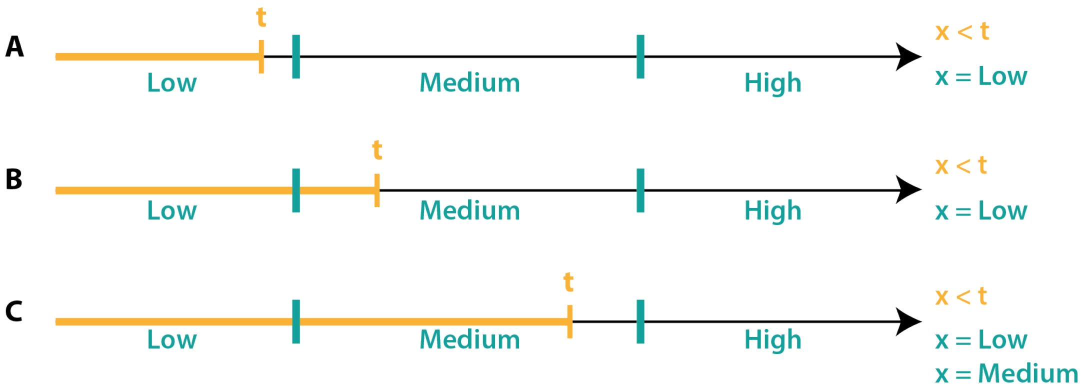

Figure S8. Discretization of variables and modification of rules. Simple example of the discretization of a uniformly distributed variable x into three levels. An original rule "$x < t$" (orange) is modified according to the number of observations in each level included in the sample support of the rule (new rule-s in green). B/ A minority of samples in the "Medium" level were included in the original sample support defined by "$x < t$", therefore the "Medium" level is not selected to make a new rule as in C/.
