# Supplementary material for: Interpreting tree ensemble machine learning models with endoR
Source: PLoS Comput Biol. 2022 Dec 14;18(12):e1010714. doi: 10.1371/journal.pcbi.1010714 (PMC9797088; doi:10.1371/journal.pcbi.1010714)
Supplement: S9 Fig — Average (line) and standard deviation (area) of identified true positive (TP) for a given number of false positive (FP). The average numbers of TP and FP in the endoR final decision ensemble are indicated with points. A, C: correspond to single variables and B, D: to pairs of variables across 100 replicates of fully simulated data. A, B/ the truncated lines of absolute numbers of TP and FP are displayed, dashed grey lines denote the ground truth number of TP. C, D/ the full curves of TP and FP rates are displayed. Lines are dashed when necessary due to overlaps. ‘Random’ signifies results expected with a randomization null model. A, C/ All methods almost identified only TP first and then FP. B, D/ endoR better discriminated TP from FP edges than SHAP. Only endoR does not return all features and interactions, hence limiting the number of FPs in the final decision ensembles, although resulting in lower recall too. (PDF) [file pcbi.1010714.s013.pdf]

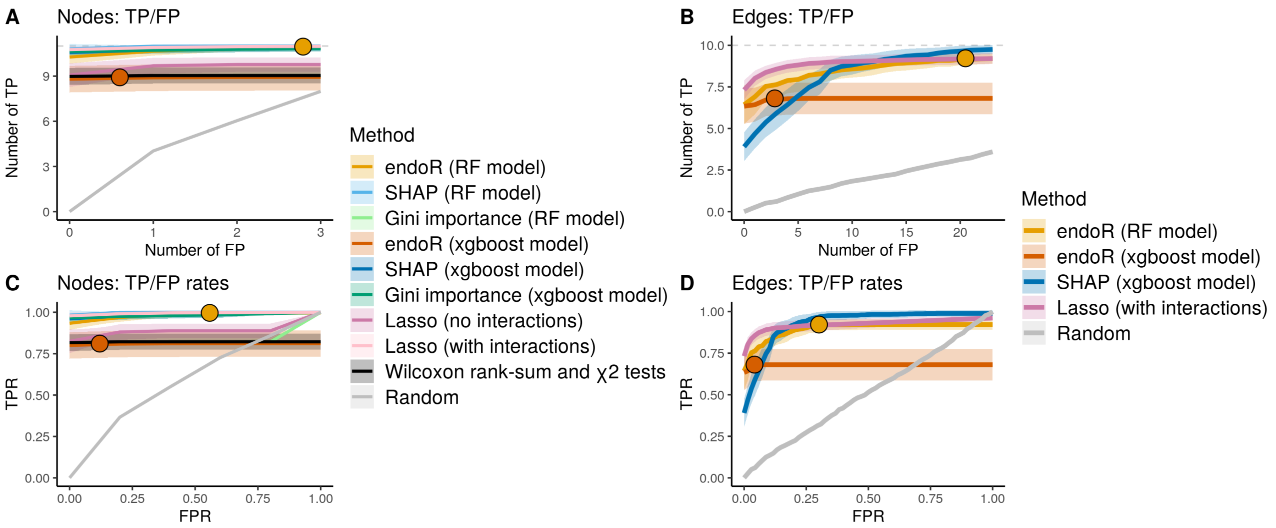

Figure S9. endoR performs as well as state-of-the-art methods at identifying variables and pairs of variables predictive of a target from fully simulated data. Average (line) and standard deviation (area) of identified true positive (TP) for a given number of false positive (FP). The average numbers of TP and FP in the endoR final decision ensemble are indicated with points. A, C: correspond to single variables and B, D: to pairs of variables across 100 replicates of fully simulated data. A, B/ the truncated lines of absolute numbers of TP and FP are displayed, dashed grey lines denote the ground truth number of TP. C, D/ the full curves of TP and FP rates are displayed. Lines are dashed when necessary due to overlaps. 'Random' signifies results expected with a randomization null model. A, C/ All methods almost identified only TP first and then FP. B, D/ endoR better discriminated TP from FP edges than SHAP. Only endoR does not return all features and interactions, hence limiting the number of FPs in the final decision ensembles, although resulting in lower recall too.
