# Supplementary material for: Interpreting tree ensemble machine learning models with endoR
Source: PLoS Comput Biol. 2022 Dec 14;18(12):e1010714. doi: 10.1371/journal.pcbi.1010714 (PMC9797088; doi:10.1371/journal.pcbi.1010714)
Supplement: S10 Fig — SHAP values were calculated from the random forest classifier trained to predict an artificial phenotype simulated from real metagenomes (n = 2147, p = 520 taxa; see Fig 2). A/ The feature importance is given by the average of the absolute SHAP values across samples and is plotted for each sample as well. B/ The SHAP interaction values could not be calculated due to the lack of R-implementation for calculating SHAP interactions from random forests. Consequently, we plotted SHAP values of the four taxa with the highest feature importances (y-axis) according to taxa relative abundances (log10 transformed, x-axis) and colored points by each of the four group category (pink: sample from the group category indicated in the plot title, blue: sample from the other categories). We note that this method of analysis does not scale well as the number of features increases. (PDF) [file pcbi.1010714.s014.pdf]

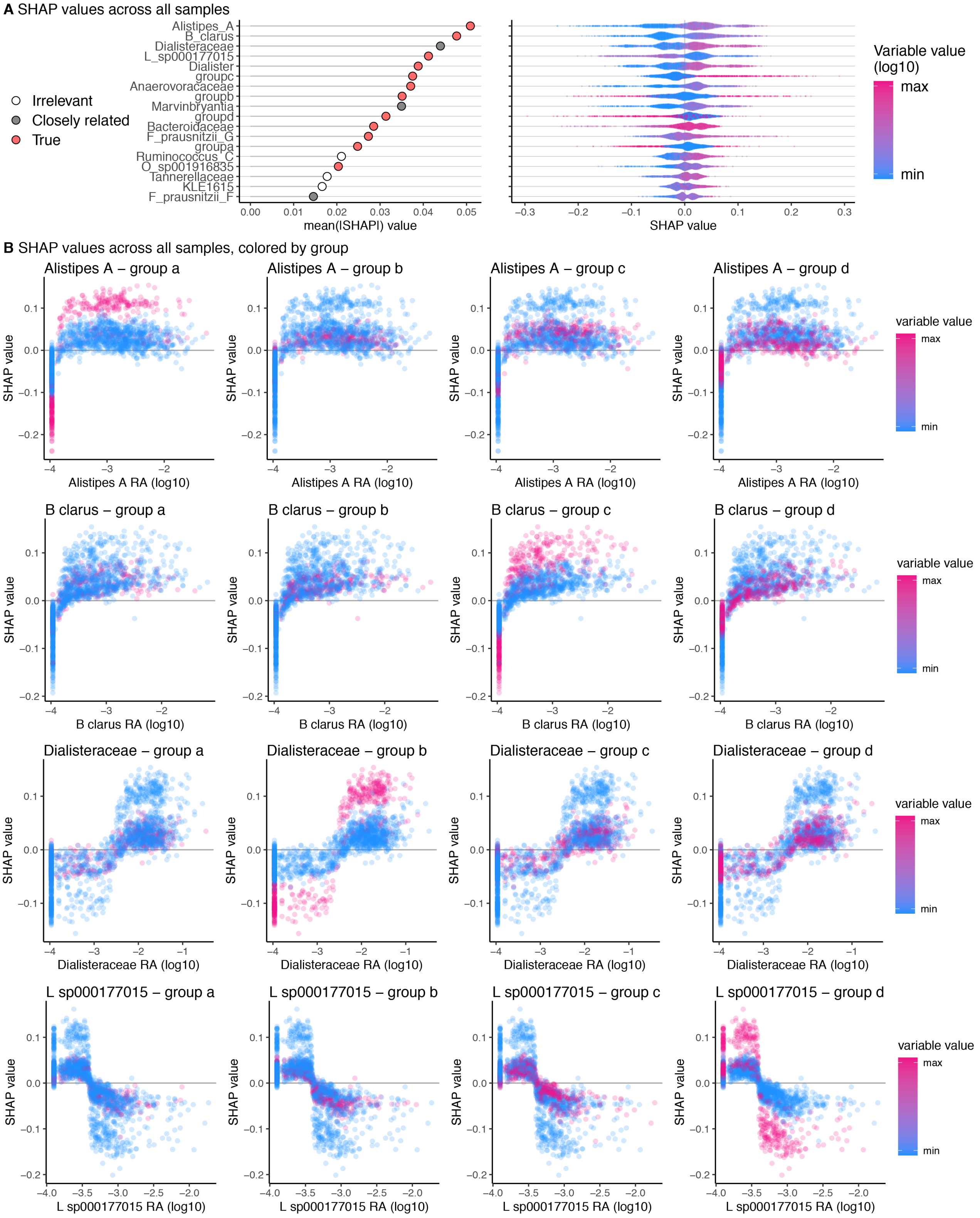

Figure S10. SHAP values from the RF classifier SHAP values were calculated from the random forest classifier trained to predict an artificial phenotype simulated from real metagenomes ($n = 2147$, $p = 520$ taxa; see Figure 2). A/ The feature importance is given by the average of the absolute SHAP values across samples and is plotted for each sample as well. B/ The SHAP interaction values could not be calculated due to the lack of R-implementation for calculating SHAP interactions from random forests. Consequently, we plotted SHAP values of the four taxa with the highest feature importances (y-axis) according to taxa relative abundances (log10 transformed, x-axis) and colored points by each of the four group category (pink: sample from the group category indicated in the plot title, blue: sample from the other categories). We note that this method of analysis does not scale well as the number of features increases.
