# Supplementary material for: Interpreting tree ensemble machine learning models with endoR
Source: PLoS Comput Biol. 2022 Dec 14;18(12):e1010714. doi: 10.1371/journal.pcbi.1010714 (PMC9797088; doi:10.1371/journal.pcbi.1010714)
Supplement: S11 Fig — SHAP values were calculated from the XGBoost classifier trained to predict an artificial phenotype simulated from real metagenomes (n = 2147, p = 520 taxa; see Fig 2). A/ The feature and interaction importances are given by the average of the absolute SHAP values across samples. B/ Given the high number of features and interactions, we only plotted the top five feature importances of single variables and top nine feature importances for interactions (marked with a start on A/). For single variables, the point color corresponds to the x-axis value. (PDF) [file pcbi.1010714.s015.pdf]

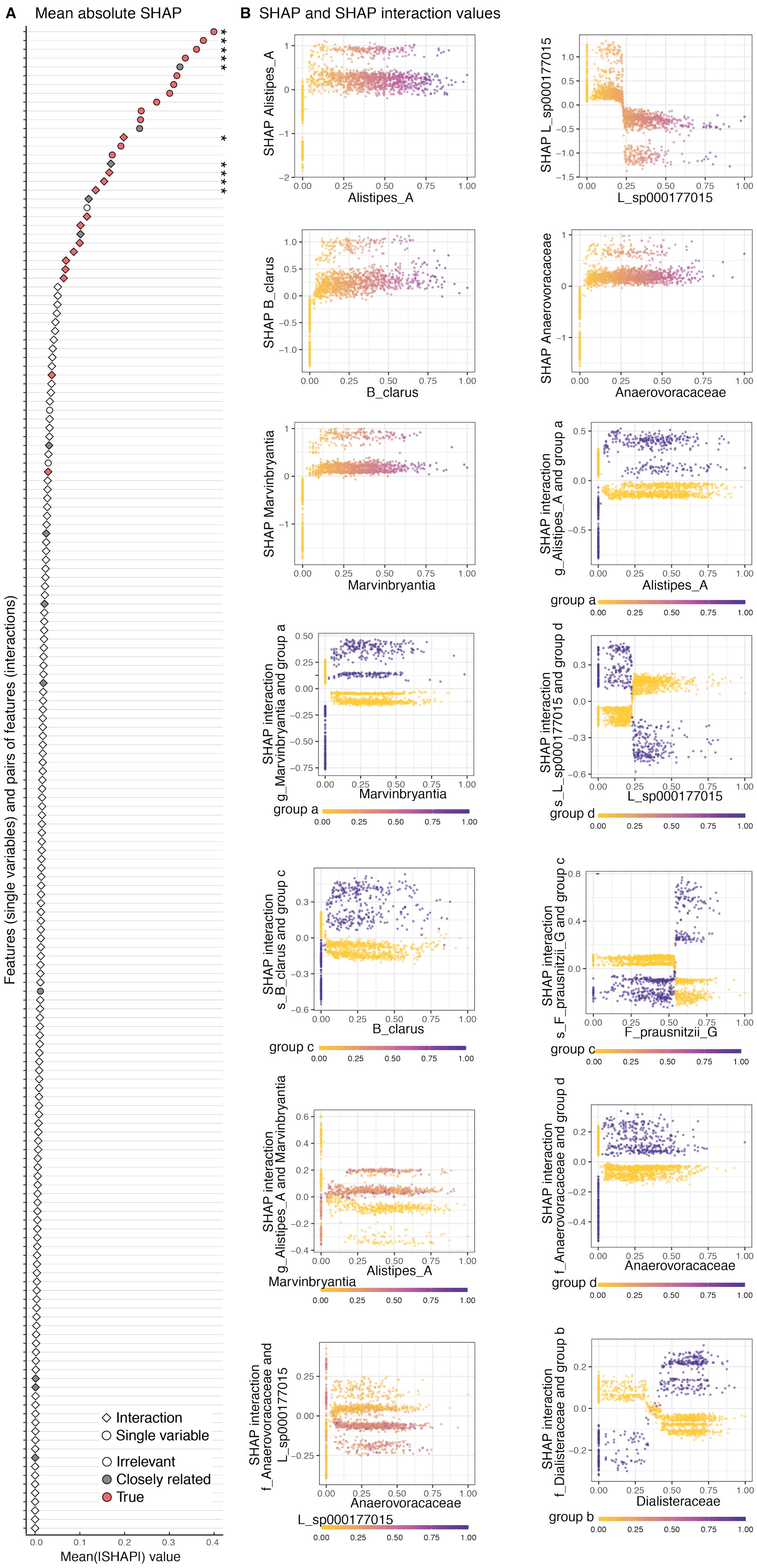

Figure S11. SHAP values from the XGBoost classifier SHAP values were calculated from the XGBoost classifier trained to predict an artificial phenotype simulated from real metagenomes ($n = 2147$, $p = 520$ taxa; see Figure 2). A/ The feature and interaction importances are given by the average of the absolute SHAP values across samples. B/ Given the high number of features and interactions, we only plotted the top five feature importances of single variables and top nine feature importances for interactions (marked with a start on A/). For single variables, the point color corresponds to the x-axis value.
