# Supplementary material for: Interpreting tree ensemble machine learning models with endoR
Source: PLoS Comput Biol. 2022 Dec 14;18(12):e1010714. doi: 10.1371/journal.pcbi.1010714 (PMC9797088; doi:10.1371/journal.pcbi.1010714)
Supplement: S12 Fig — When the log10 of relative abundances is displayed, a pseudo-count equal to the minimal relative abundance detected in the dataset (3 ⋅ 10−7) was used to show samples for which taxa were not detected (relative abundance = 0). Boxplots and points are colored by healthy status, with healthy individuals in orange and cirrhotic ones in blue. A/ Taxonomic levels are indicated with the prefixes: ‘f_’ = family, ‘g_’ = genus, ‘s_’ = species. Taxa are organized by family taxonomic level (separated by grey lines). The background indicates whether taxa were identified in this article and the original study (red), only in this article via a RF model and endoR (green), or only in the original study (yellow). Species for which the relative abundances were not available in the published dataset (downloaded from the ML task repository) are indicated with a star. Taxa in B-I/ are indicated by an arrow. B-E/ The four taxa taxa with highest feature importance (FI) identified by endoR to classify healthy versus cirrhotic microbiomes (see Fig 5A). F-I/ Taxa exclusively identified in the original study [41] or with a random forest and endoR. (PDF) [file pcbi.1010714.s016.pdf]

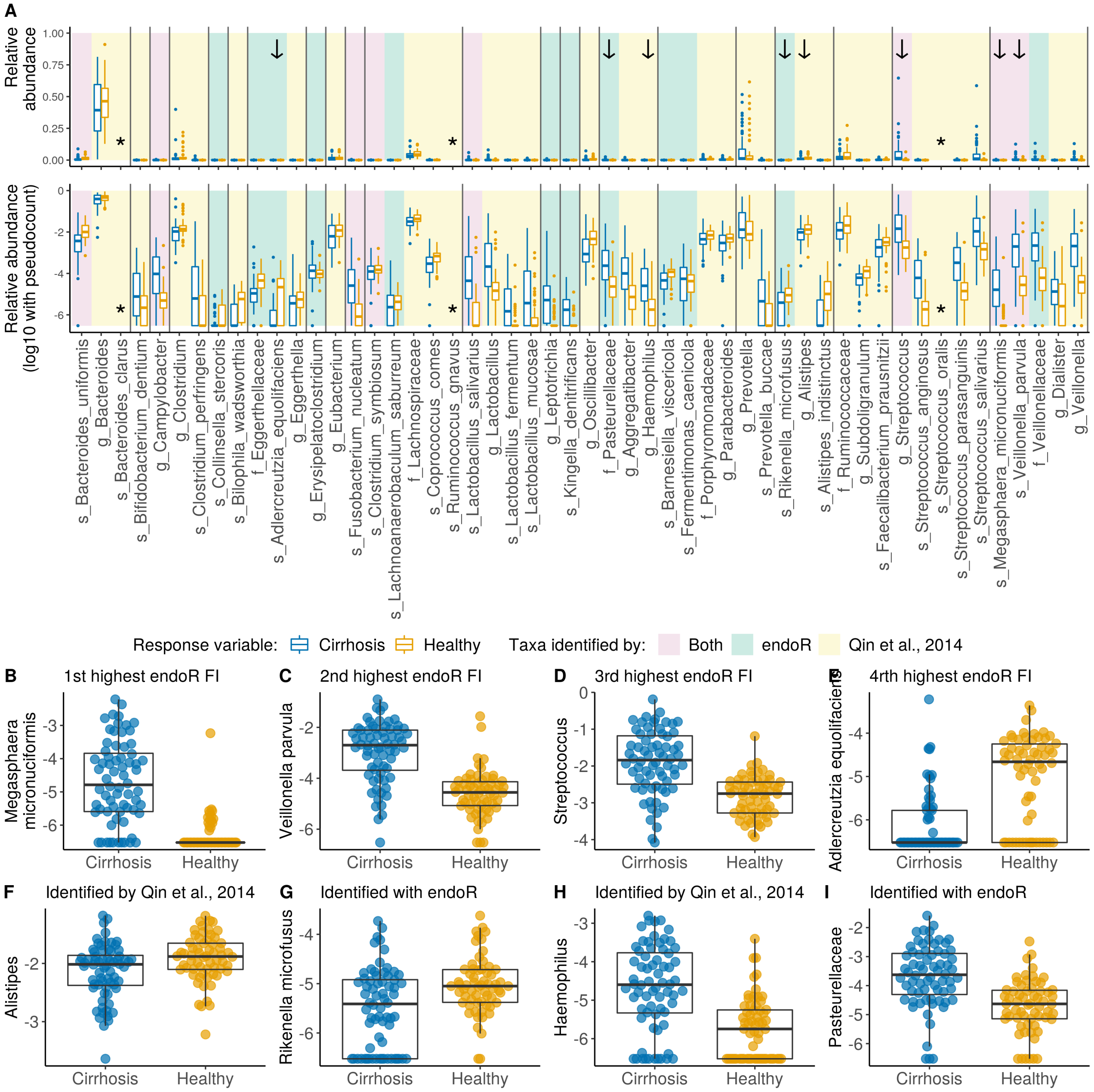

Figure S12. Relative abundances of taxa identified using a RF and endoR versus statistical tests in the original study (1). When the log10 of relative abundances is displayed, a pseudo-count equal to the minimal relative abundance detected in the dataset $(3 \cdot 10^{-7})$ was used to show samples for which taxa were not detected (relative abundance = 0). Boxplots and points are colored by healthy status, with healthy individuals in orange and cirrhotic ones in blue. A/ Taxonomic levels are indicated with the prefixes: 'f_' = family, 'g_' = genus, 's_' = species. Taxa are organized by family taxonomic level (separated by grey lines). The background indicates whether taxa were identified in this article and the original study (red), only in this article via a RF model and endoR (green), or only in the original study (yellow). Species for which the relative abundances were not available in the published dataset (downloaded from the ML task repository) are indicated with a star. Taxa in B-I/ are indicated by an arrow. B-E/ The four taxa taxa with highest feature importance (FI) identified by endoR to classify healthy versus cirrhotic microbiomes (see Fig 5A). F-I/ Taxa exclusively identified in the original study (1) or with a random forest and endoR.

References

1. Nan Qin, Fengling Yang, Ang Li, Edi Prifti, Yanfei Chen, Li Shao, Jing Guo, Emmanuelle Le Chatelier, Jian Yao, Lingjiao Wu, et al. Alterations of the human gut microbiome in liver cirrhosis. *Nature*, 513(7516):59–64, 2014.
