# Supplementary material for: Interpreting tree ensemble machine learning models with endoR
Source: PLoS Comput Biol. 2022 Dec 14;18(12):e1010714. doi: 10.1371/journal.pcbi.1010714 (PMC9797088; doi:10.1371/journal.pcbi.1010714)
Supplement: S13 Fig — A/ Features with the best Gini importance. B/ Comparison of the Gini importance and the number of times features were selected across the 10 cross-validation (CV) sets. C/ Comparison of the Gini and endoR importance. D/ Gini importance of all features selected by the taxa-aware gRRF algorithm. In all plots taxonomic levels are indicated in the labels with ‘f_’: family, ‘g_’: genus, and ‘s_’: species, and orders are indicated via point and label colors. (PDF) [file pcbi.1010714.s017.pdf]

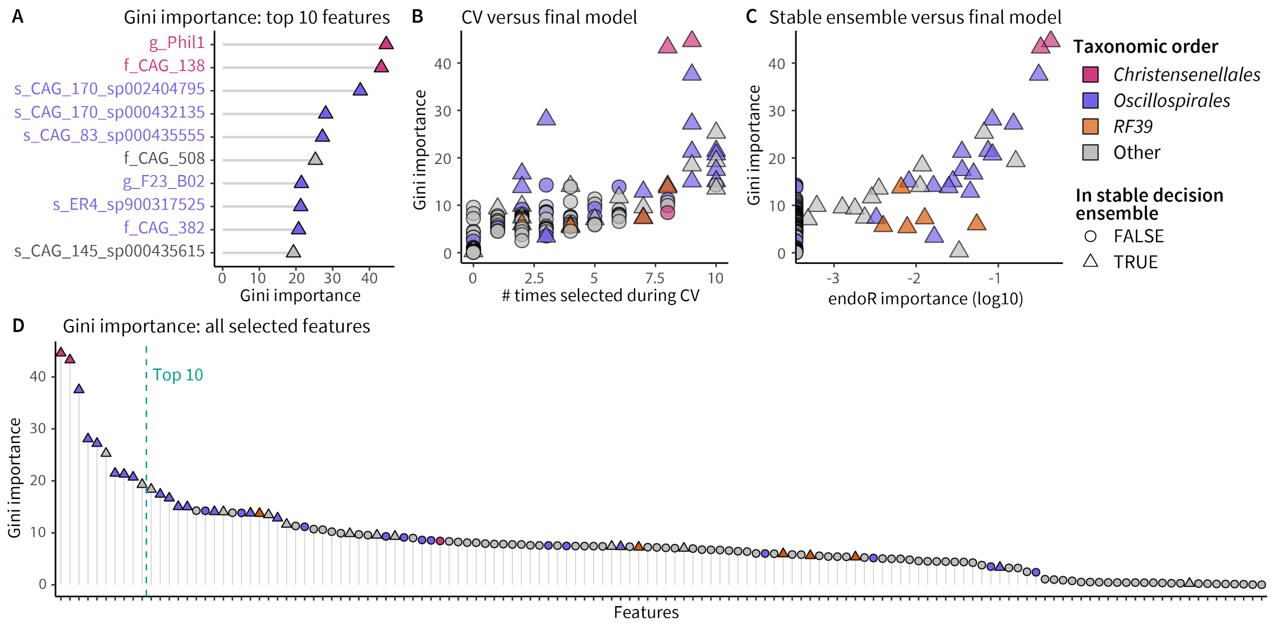

Figure S13. The Gini and endoR importances are consistent between the RF model and stable decision ensemble. A/ Features with the best Gini importance. B/ Comparison of the Gini importance and the number of times features were selected across the 10 cross-validation (CV) sets. C/ Comparison of the Gini and endoR importance. D/ Gini importance of all features selected by the taxa-aware gRRF algorithm. In all plots taxonomic levels are indicated in the labels with 'f_': family, 'g_': genus, and 's_': species, and orders are indicated via point and label colors.
