# Supplementary material for: Interpreting tree ensemble machine learning models with endoR
Source: PLoS Comput Biol. 2022 Dec 14;18(12):e1010714. doi: 10.1371/journal.pcbi.1010714 (PMC9797088; doi:10.1371/journal.pcbi.1010714)
Supplement: S14 Fig — (PDF) [file pcbi.1010714.s018.pdf]

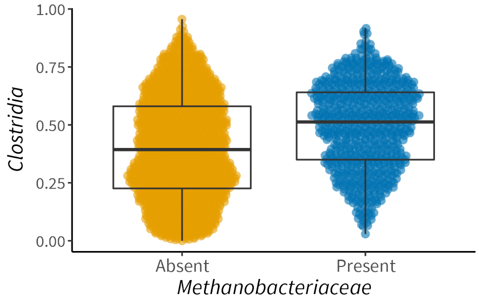

Figure S14. The relative abundance of *Clostridia* is higher in samples where *Methanobacteriaceae* are detected.
