# Supplementary material for: Interpreting tree ensemble machine learning models with endoR
Source: PLoS Comput Biol. 2022 Dec 14;18(12):e1010714. doi: 10.1371/journal.pcbi.1010714 (PMC9797088; doi:10.1371/journal.pcbi.1010714)
Supplement: S15 Fig — We looked into the genomes of representative species of taxa used to predict the presence/absence of Methanobacteriaceae in human guts microbiome from 2203 individuals for involved in H2 metabolism. The number of copies of genes involved in the following pathways or function were counted: sulfate reduction (SRB): dsrA and dsrB genes [82]; acetogenesis (Acetogen): fhs gene [83]; H2 production, uptake and sensing as determined by the HydDB database [84]. At the genus and family taxonomic levels, we used the average number of copies across species from the given level and weighted the number of copies of each species by the average relative abundance of species in the dataset. Accordingly, if the most abundant species of a specific genus had high number of gene copies, the number of copies for that genus would also be high. When genes were grouped by general function, we summed the number of copies (e.g., the SRB gene copy number corresponds to the sum of gene copies of dsrA and dsrB). A/ The ratios of gene copy number by genome size for each of the endoR selected features are consistent with the absolute number of copies displayed in Fig 6B. For each representative species, the number of gene copies was divided by the genome size. General functions and genes are displayed and separated by black lines (blocks of genes with the same general function), general functions are separated from specific genes by a grey line. B/ Number of genes copies from each group for taxa selected by feature selection. C/ Occurrence of genes from each group across all taxonomic features used to train models to predict the occurrence of Methanobacteriaceae in human guts. (PDF) [file pcbi.1010714.s019.pdf]

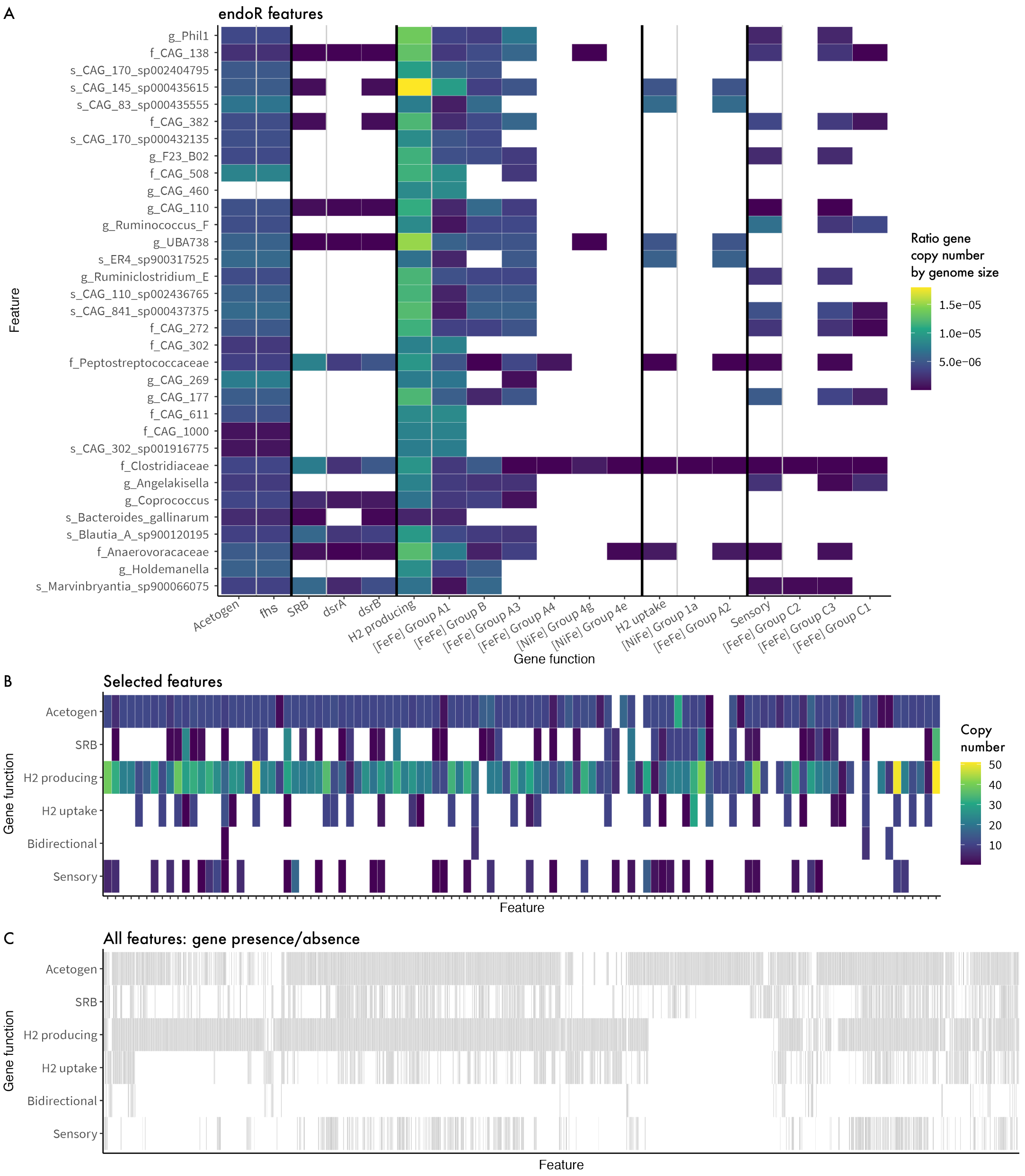

Figure S15. Copy number of genes involved in H_2 consumption and production across taxa used to predict the presence/absence of *Methanobacteriaceae*. We looked into the genomes of representative species of taxa used to predict the presence/absence of *Methanobacteriaceae* in human guts microbiome from 2203 individuals for involved in H_2 metabolism. The number of copies of genes involved in the following pathways or function were counted: sulfate reduction (SRB): *dsrA* and *dsrB* genes (1); acetogenesis (Acetogen): *fhs* gene (2); H_2 production, uptake and sensing as determined by the HydDB database (3). At the genus and family taxonomic levels, we used the average number of copies across species from the given level and weighted the number of copies of each species by the average relative abundance of species in the dataset. Accordingly, if the most abundant species of a specific genus had high number of gene copies, the number of copies for that genus would also be high. When genes were grouped by general function, we summed the number of copies (e.g., the SRB gene copy number corresponds to the sum of gene copies of *dsrA* and *dsrB*). A/ The ratios of gene copy number by genome size for each of the endoR selected features are consistent with the absolute number of copies displayed in Fig 6B. For each representative species, the number of gene copies was divided by the genome size. General functions and genes are displayed and separated by black lines (blocks of genes with the same general function), general functions are separated from specific genes by a grey line. B/ Number of genes copies from each group for taxa selected by feature selection. C/ Occurrence of genes from each group across all taxonomic features used to train models to predict the occurrence of *Methanobacteriaceae* in human guts.

References

1. Jordan A Fish, Benli Chai, Qiong Wang, Yanni Sun, C Titus Brown, James M Tiedje, and James R Cole. Fungene: the functional gene pipeline and repository. *Frontiers in microbiology*, 4:291, 2013.
2. Abhijeet Singh, Bettina Müller, Hans-Henrik Fuxelius, and Anna Schnürer. Acetobase: a functional gene repository and database for formyltetrahydrofolate synthetase sequences. *Database*, 2019, 2019.
3. Dan Søndergaard, Christian NS Pedersen, and Chris Greening. Hyddb: a web tool for hydrogenase classification and analysis. *Scientific reports*, 6(1):1–8, 2016.
