# Supplementary material for: Interpreting tree ensemble machine learning models with endoR
Source: PLoS Comput Biol. 2022 Dec 14;18(12):e1010714. doi: 10.1371/journal.pcbi.1010714 (PMC9797088; doi:10.1371/journal.pcbi.1010714)
Supplement: S16 Fig — Each plot illustrates the support of a decision D in the feature space spanned by variables {xj, xk}, i.e., the values that the decision can take on variables xj and xk. A/ Original decision D. B/ Modified decision Djrm resulting from removing variable xj from decision D. C/ Modified decision Dj,krm resulting from removing variables xj and xk from decision D. A-C/ The support SD of the originl decision is indicated by the stripped areas, such as samples in the support of D all take positive values on xj and xk. The support of each decision, i.e., SD, SDjrm and SDj,krm for A, B, and C, respectively, is visualized by the colored region. B/ When we remove variable xj from the rule rD of D, the support SDjrm is extended to samples taking negative values on xj (colored area). C/ Similarly, when we remove a pair of variables {xj, xk} from rD, samples in SDj,krm can take positive and negative values on j and k. For SDjrm and SDj,krm, we calculate y^Djrm and y^Dj,krm, respectively, using all samples in SDjrm and SDj,krm. The decision-wise importance δDj of j in D is calculated by comparing the error of y^Djrm on SD (B/) versus the error of y^D on SD (A/). Similarly, to calculate the decision-wise importance of a pair of variables {j, k} in a decision D, we compare the error from the decision not constraining values on j or k, with y^Dj,krm on SD (C/) to the error of the decision with y^D on SD (A/). (PDF) [file pcbi.1010714.s020.pdf]

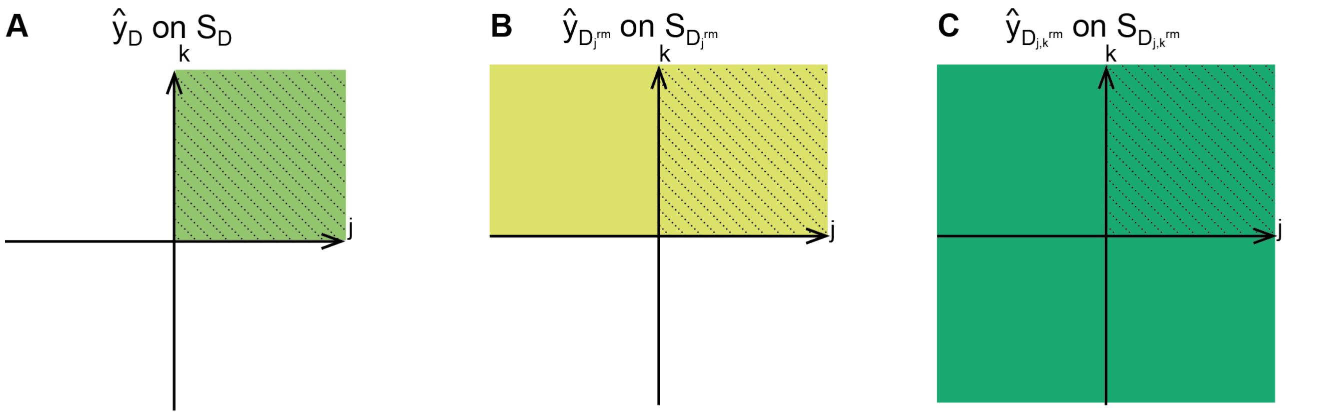

Figure S16. Visualization of how decisions are modified to calculate the importance of variables. Each plot illustrates the support of a decision D in the feature space spanned by variables $\{x^j, x^k\}$, i.e., the values that the decision can take on variables x^j and x^k. A/ Original decision D. B/ Modified decision D_j^{rm} resulting from removing variable x^j from decision D. C/ Modified decision $D_{j,k}^{\text{rm}}$ resulting from removing variables x^j and x^k from decision D. A-C/ The support S_D of the originl decision is indicated by the stripped areas, such as samples in the support of D all take positive values on x^j and x^k. The support of each decision, i.e., S_D, $S_{D_j^{\text{rm}}}$ and $S_{D_{j,k}^{\text{rm}}}$ for A, B, and C, respectively, is visualized by the colored region. B/ When we remove variable x^j from the rule r_D of D, the support $S_{D_j^{\text{rm}}}$ is extended to samples taking negative values on x^j (colored area). C/ Similarly, when we remove a pair of variables $\{x^j, x^k\}$ from r_D, samples in $S_{D_{j,k}^{\text{rm}}}$ can take positive and negative values on j and k. For $S_{D_j^{\text{rm}}}$ and $S_{D_{j,k}^{\text{rm}}}$, we calculate $\hat{y}_{D_j^{\text{rm}}}$ and $\hat{y}_{D_{j,k}^{\text{rm}}}$, respectively, using all samples in $S_{D_j^{\text{rm}}}$ and $S_{D_{j,k}^{\text{rm}}}$. The decision-wise importance δ_D^j of j in D is calculated by comparing the error of $\hat{y}_{D_j^{\text{rm}}}$ on S_D (B/) versus the error of $\hat{y}_D$ on S_D (A/). Similarly, to calculate the decision-wise importance of a pair of variables $\{j, k\}$ in a decision D, we compare the error from the decision not constraining values on j or k, with $\hat{y}_{D_{j,k}^{\text{rm}}}$ on S_D (C/) to the error of the decision with $\hat{y}_D$ on S_D (A/).
