# Supplementary material for: Interpreting tree ensemble machine learning models with endoR
Source: PLoS Comput Biol. 2022 Dec 14;18(12):e1010714. doi: 10.1371/journal.pcbi.1010714 (PMC9797088; doi:10.1371/journal.pcbi.1010714)
Supplement: S17 Fig — (PDF) [file pcbi.1010714.s021.pdf]

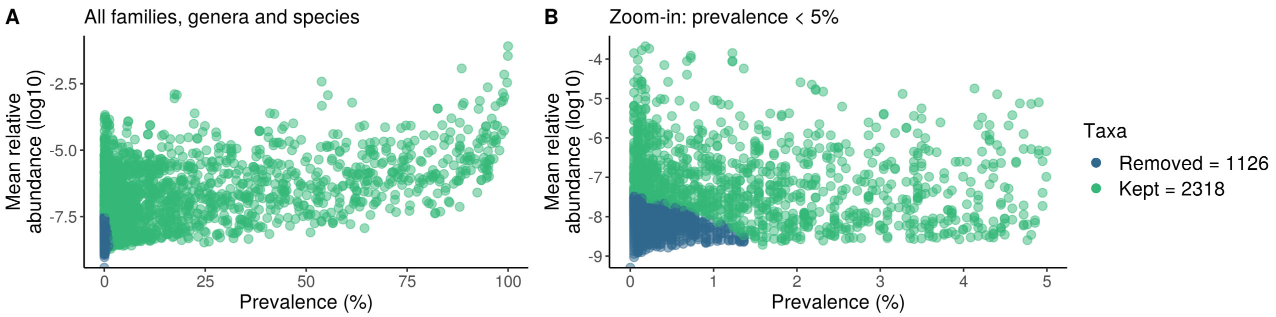

Figure S17. Mean relative abundances and prevalence of family, genus and species taxonomic levels in the metagenomic data.
