# Supplementary material for: Interpreting tree ensemble machine learning models with endoR
Source: PLoS Comput Biol. 2022 Dec 14;18(12):e1010714. doi: 10.1371/journal.pcbi.1010714 (PMC9797088; doi:10.1371/journal.pcbi.1010714)
Supplement: S18 Fig — A-C, H-K/ Principal coordinate analysis ordination of the Jensen-Shannon distance matrix computed from genera relative abundances. A-B/ Samples colored by relative abundance (RA) of Bacteroides and Prevotella, respectively. To calculate the log, a pseudocount equal to the minimal non-null RA was given to samples for which the genus was not detected, i.e., RA = 0. C/ Samples colored by enterotype cluster [2, 64]; ETF: Firmicutes, ETB: Bacteroides, ETP: Prevotella; colors correspond to those on E. J-K/ Samples are colored by their country of origin grouped by region when possible (e.g., Canada and USA grouped into North America; S7 Table), and points are emphasized (larger and less transparent) if they were sampled from non-westernized (J) or westernized (K) populations. D-G/ Average silhouette score (bar) within each k-mean cluster computed from the Jensen-Shannon distance matrix and across clusters (thick line). Dashed line: threshold above which clustering strength is moderate. (PDF) [file pcbi.1010714.s022.pdf]

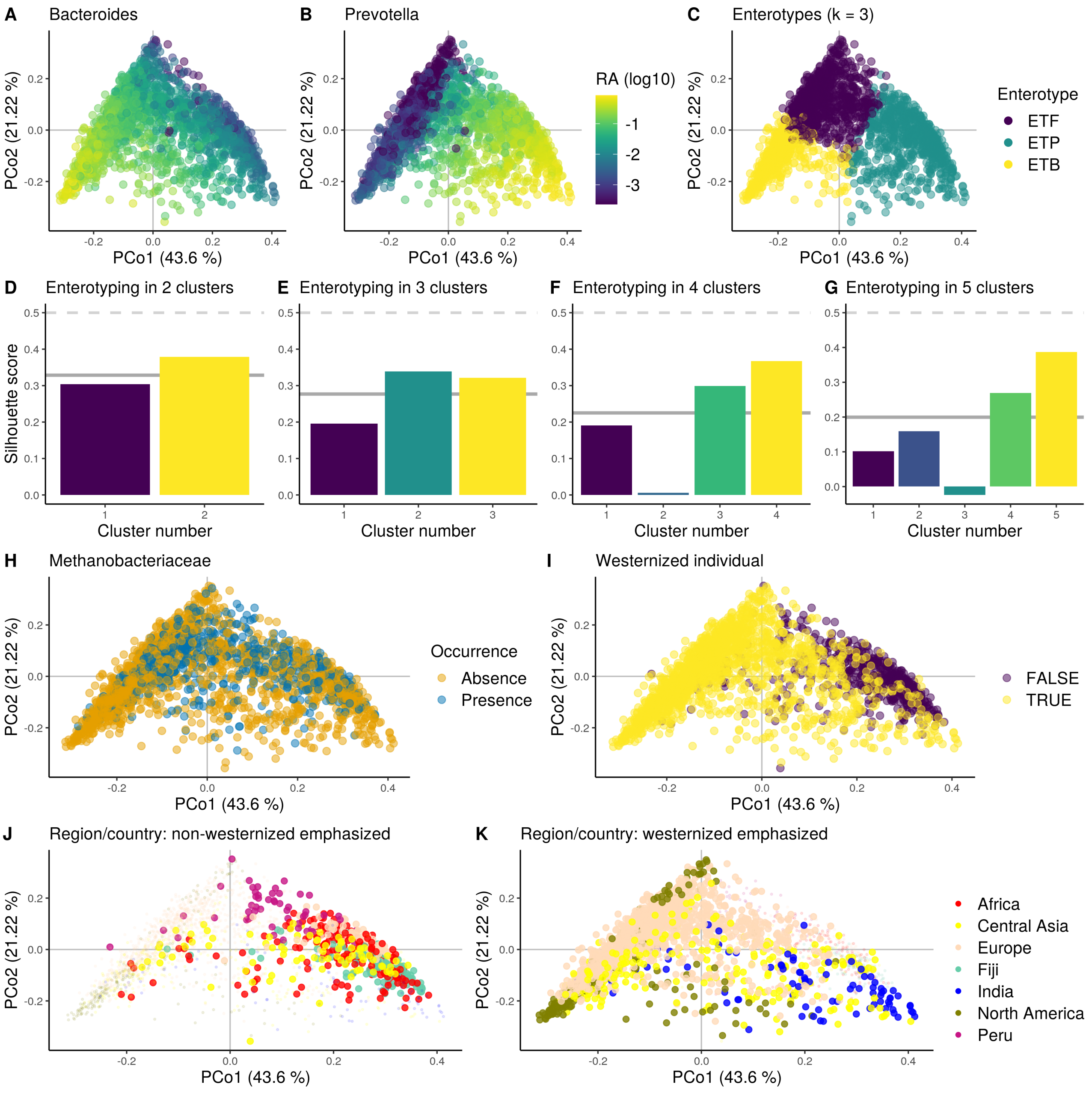

Figure S18. Gut microbiota of a large cohort of healthy individuals (n = 2203 individuals) approximately segregate along the enterotype landscape. A-C, H-K/ Principal coordinate analysis ordination of the Jensen-Shannon distance matrix computed from genera relative abundances. A-B/ Samples colored by relative abundance (RA) of *Bacteroides* and *Prevotella*, respectively. To calculate the log, a pseudocount equal to the minimal non-null RA was given to samples for which the genus was not detected, i.e., RA = 0. C/ Samples colored by enterotype cluster (1, 2); ETF: *Firmicutes*, ETB: *Bacteroides*, ETP: *Prevotella*; colors correspond to those on E. J-K/ Samples are colored by their country of origin grouped by region when possible (e.g., Canada and USA grouped into North America; Table S7), and points are emphasized (larger and less transparent) if they were sampled from non-westernized (J) or westernized (K) populations. D-G/ Average silhouette score (bar) within each k-mean cluster computed from the Jensen-Shannon distance matrix and across clusters (thick line). Dashed line: threshold above which clustering strength is moderate (3).

References

1. Manimozhiyan Arumugam, Jeroen Raes, Eric Pelletier, Denis Le Paslier, Takuji Yamada, Daniel R Mende, Gabriel R Fernandes, Julien Tap, Thomas Bruls, Jean-Michel Batto, Marcelo Bertalan, Natalia Borruel, Francesc Casellas, Leyden Fernandez, Laurent Gautier, Torben Hansen, Masahira Hattori, Tetsuya Hayashi, Michiel Kleerebezem, Ken Kurokawa, Marion Leclerc, Florence Levenez, Chaysavanh Manichanh, H. Bjørn Nielsen, Trine Nielsen, Nicolas Pons, Julie Poulain, Junjie Qin, Thomas Sicheritz-Ponten, Sebastian Tims, David Torrents, Edgardo Ugarte, Erwin G Zoetendal, Jun Wang, Francisco Guarner, Oluf Pedersen, Willem M. de Vos, Søren Brunak, Joel Doré, Jean Weissenbach, S Dusko Ehrlich, and Peer Bork. Enterotypes of the human gut microbiome. *Nature*, 473 (7346):174–180, 5 2011. ISSN 0028-0836. doi: 10.1038/nature09944.
2. Paul I Costea, Falk Hildebrand, Manimozhiyan Arumugam, Fredrik Bäckhed, Martin J Blaser, Frederic D Bushman, Willem M De Vos, S Dusko Ehrlich, Claire M Fraser, Masahira Hattori, et al. Enterotypes in the landscape of gut microbial community composition. *Nature microbiology*, 3(1):8–16, 2018.
3. Omry Koren, Dan Knights, Antonio Gonzalez, Levi Waldron, Nicola Segata, Rob Knight, Curtis Huttenhower, and Ruth E. Ley. A Guide to Enterotypes across the Human Body: Meta-Analysis of Microbial Community Structures in Human Microbiome Datasets. *PLoS Computational Biology*, 9(1):e1002863, 1 2013. ISSN 1553-7358. doi: 10.1371/journal.pcbi.1002863.
