# Supplementary material for: Interpreting tree ensemble machine learning models with endoR
Source: PLoS Comput Biol. 2022 Dec 14;18(12):e1010714. doi: 10.1371/journal.pcbi.1010714 (PMC9797088; doi:10.1371/journal.pcbi.1010714)
Supplement: Attachment — Submitted filename: response_to_reviewers.pdf [file pcbi.1010714.s036.pdf]

Dear Dr. Youngblut,

Thank you very much for submitting your manuscript "Interpreting tree ensemble machine learning models with endoR" for consideration at PLOS Computational Biology.

As with all papers reviewed by the journal, your manuscript was reviewed by members of the editorial board and by several independent reviewers. In light of the reviews (below this email), we would like to invite the resubmission of a significantly-revised version that takes into account the reviewers' comments.

The reviewers generally consider that the work is a good contribution to the field and the manuscript is well-written. However, they also raise some points that should be addressed. In particular, the concern of reviewer #1 as to how the feature selection is performed is most worrisome as it is an issue of correctness.

We cannot make any decision about publication until we have seen the revised manuscript and your response to the reviewers' comments. Your revised manuscript is also likely to be sent to reviewers for further evaluation.

When you are ready to resubmit, please upload the following:

[1] A letter containing a detailed list of your responses to the review comments and a description of the changes you have made in the manuscript. Please note while forming your response, if your article is accepted, you may have the opportunity to make the peer review history publicly available. The record will include editor decision letters (with reviews) and your responses to reviewer comments. If eligible, we will contact you to opt in or out.

[2] Two versions of the revised manuscript: one with either highlights or tracked changes denoting where the text has been changed; the other a clean version (uploaded as the manuscript file).

Important additional instructions are given below your reviewer comments.

Please prepare and submit your revised manuscript within 60 days. If you anticipate any delay, please let us know the expected resubmission date by replying to this email. Please note that revised manuscripts received after the 60-day due date may require evaluation and peer review similar to newly submitted manuscripts.

Thank you again for your submission. We hope that our editorial process has been constructive so far, and we welcome your feedback at any time. Please don't hesitate to contact us if you have any questions or comments.

Sincerely,

Luis Pedro Coelho
Associate Editor
PLOS Computational Biology

Dina Schneidman
Software Editor
PLOS Computational Biology

****

The reviewers generally consider that the work is a good contribution to the field and the manuscript is well-written. However, they also raise some points that should be addressed. In particular, the concern of reviewer #1 as to how the feature selection is performed is most worrisome as it is an issue of correctness.

Reviewer's Responses to Questions

Comments to the Authors:
Please note here if the review is uploaded as an attachment.

We thank all reviewers and the editor for their valuable comments on our manuscript. We have now carefully prepared a revision of our work. Some of the major changes include the following points:

1. **We have clarified our methodology for feature selection.**
2. **We have included LASSO models and other competing methods previously not evaluated in this work.**
3. **We have added an assessment of the usefulness of global null models.**
4. **All ready-to-use data are now available on FigShare.**
5. **The endoR R package has been submitted to CRAN.**

Below, we provide a detailed point-by-point response addressing the issues raised. We feel that the comments have helped to significantly improve the quality of the paper. Unless stated otherwise, all numbers and references refer to the new version of the manuscript.

Reviewer #1

Major comments

Ruaud and colleagues present endoR, an R package aimed to enable better interpretation of tree ensemble methods, particularly for microbiome data. They showcase the utility of their package using fully simulated data, artificial phenotypes (real data with a simulated response variable), and two real-world applications (prediction of liver cirrhosis from fecal metagenomes and prediction of the presence of Methanobacteriaceae in the gut). The software addressed a well-known problem in the field (i.e. limited interpretability of complex tree-ensemble machine learning methods) and their article is well written (albeit a bit verbose in places), but several questions remains, especially regarding feature selection, comparisons to other tools/measures and comparisons to other machine learning methods.

We thank the reviewer for their assessment of our work. We have responded to each of the raised points individually below.

Feature selection: It sounds like the feature selection step was performed before model training (I hope I did not miss a detailed description of the feature selection, but in any case I would advise the authors to describe their feature selection procedure more prominently and in more detail). This can lead to overfitting and over-optimistic models and should be avoided (see for example https://www.ncbi.nlm.nih.gov/pubmed/19880370).

We performed feature selection during model training (Supplementary Figure 12-A). To be more precise, the fitted models we consider are a combination of feature selection with a subsequent random forest (RF). As with the hyperparameters of the RF

(e.g., number of trees), the type of feature selection is selected using cross-validation (CV), hence avoiding overfitting. This was previously described in Supplementary Figure 12-A. In the updated manuscript, we have tried to clarify this at the following points in the text:

- **In the 3rd and 4th paragraphs of the Introduction (on page 1, 2nd column), we now more clearly separate feature selection as a regularization step in a prediction model from feature importance methods which are interpretation tools.**
- **In the section "Interpreting tree ensemble models with endoR" (2nd paragraph on page 3), we are now more precise about the fact that the feature selection is part of the model fitting and explicitly refer to the supplementary figure for a detailed explanation.**
- **When describing the fitted models for the three applications (Methods sections for: artificial phenotypes, cirrhosis study and *Methanobacteriaceae*, pages 15 and 16), we have clarified the feature selection step further.**
- **Finally, we have updated the descriptions of the feature selection in the Supplementary Methods (page 24) and added references to Supplementary Figure 12-A when appropriate.**

Comparison to other methods: The comparison to other methods (Gini coefficient, SHAP values, etc.) is the part of the manuscript that could be improved the most.

We thank the reviewer for this suggestion. We have now included the LASSO method (models with and without pairwise interactions) and additionally processed the FSD with the competing methods. Please also see our comments below for more information related to this topic.

Figure 2G indicates that endoR might perform better than the Gini coefficient, but it is only shown for the top features in the main text.

Figure 2G shows the feature importance for all 18 features in the model: due to feature selection, not all taxa in the dataset were included, and their feature importance can be considered as null for any interpretation method applied to the random forest (e.g., endoR and Gini).

The Figure 2 caption has now been updated to explain this (page 4).

Similarly, Figure 4 focuses on the top 5 false positive features only (disregarding the reconstructed network for now). I think it would be possible to construct AUROC measures for how well Gini coefficients, SHAP values, and endoR can recover the true underlying features (in repeated(?, see below) AP simulations), covering all features by evaluating the true positive and false positive rates, even though closely related features would have to be disregarded or evaluated separately.

Figure 4 shows the number of TPs and FPs: all interpretation methods of ML models performed quite well at finding the TPs (reflecting the accuracy of the random

forest they were interpreting), and so their curves flatten once all TP have been found. Consequently, we chose to focus on the ranking of features with highest importances only, in order to better illustrate the small differences that may occur.

We have included an experiment comparing the TP/FP variables and pairwise interactions of the same competing methods when applied to the 100 FSDs (see Supplementary Figure 7, page 34).

Additionally, we have added TP/FP rates curves for both APs and FSDs in Supplementary Figure 8 (page 35).

Comparison to other machine learning methods: There are also other machine learning algorithms except the random forest routinely used in the microbiome field. For example, both LASSO or Elastic Net can have similar (or even slightly better) performance to the random forest (https://pubmed.ncbi.nlm.nih.gov/33785070/, given properly normalised input data), and both of these do not suffer of limited interpretability. The authors should compare their endoR importance measures also against features weights derived from those more white-box models.

We have added the LASSO method to our comparisons (Figure 4 for the artificial phenotypes, page 7, and Supplementary Figure 8, page 34, for the fully simulated data). Briefly, data were transformed using the centered-log ratio transformation to account for compositionality, and two LASSO models were fitted with tuning the lamba hyperparameter. The first model did not include interactions (so only variable main effects), while the second was fitted with all variables and pairwise interactions. Variables were ranked based on their absolute weight in the final model; for the model with interactions, we used the sum of the main effect and interactions weights for each variable. As we show in Supplementary Figure 8, LASSO performed as well as other methods on our simple FSD simulations, but the model was inaccurate on the APs, as shown on Figure 4. Thus, such white-box models may not capture complex interactions in microbiome sequence datasets, explaining the popularity and relatively accuracy of tree ensemble methods (random forests and gradient boosted models) for microbiome data (Marcos-Zambrano et al. 2021; Zhou and Gallins 2019; Vangay, Hillmann, and Knights 2019; Topçuoğlu et al. 2020).

The Methods (page 17) and Supplementary Methods (page 26) were updated accordingly.

Limited interactions in cirrhosis predictions: For the cirrhosis dataset, almost no meaningful interaction between features has been detected by endoR. I think the authors should discuss this observation. Does this mean that interaction networks between features do not play a prominent role in disease predictions? Relating to the point above, would a less-complex machine learning algorithm (such as LASSO) have been sufficient? (This is probably outside the scope of this paper, but how do different diseases compare in this aspect?)

Based on our findings, we agree with the reviewer that, for this dataset, “interaction networks between features do not play a prominent role in disease predictions”. To investigate this, we have fitted a LASSO model with no interactions and

using CV to select the penalty parameter. This resulted in a fairly high accuracy of 80.79%, which was still lower compared to our RF model (86.58% accuracy). These findings suggest that large parts of the signal can be explained with simple linear effects and do not require interactions. We have added a comment on this in the manuscript (last paragraph of the “Cirrhosis” section, page 9).

While this data set consists of a simple signal, this is by no means guaranteed, and we believe that more complex models have the advantage of adapting more easily to the data and potentially also picking up more complex signals, notably from modern datasets with larger sample sizes. This notion is supported by recent publications (Marcos-Zambrano et al. 2021; Zhou and Gallins 2019; Vangay, Hillmann, and Knights 2019; Topçuoğlu et al. 2020).

We have updated the Results (last paragraph of the section “endoR rediscovers previously …” on page 9) and the Methods (page 16) accordingly.

Minor comments

The prevalence threshold of 25% seems rather high. Can the authors motivate the level of the cutoff? For example, one of the best-known predictors for colorectal cancer, Fusobacterium nucleatum, is not present at a prevalence higher than 10% in a typical case-control study, and therefore, a prevalence cutoff of 5% might be more appropriate.

We agree that a 25 % cutoff is rather high, which is why we only used this cutoff for the artificial phenotypes. Our motivation was to make sure that the taxa we randomly picked to create the artificial phenotypes were prevalent enough to make rules that could apply to most samples.

For the real-world datasets (i.e., the cirrhosis and *Methanobacteriaceae* datasets), we used a progressive (or gradient) cutoff, balancing between the prevalence and mean relative abundance (see the Supplementary Methods section “Progressive filtering of rare taxa” on page 24). In this manner, highly prevalent taxa with very low relative abundance and rare taxa with high relative abundance (when present) were kept in the set of features.

As discussed in the literature (Schloss 2020; Nearing et al. 2022; Neu, Allen, and Roy 2021; Weiss et al. 2015), microbial taxon abundance/prevalence cutoffs are rather arbitrary and depend on specific factors inherent to the particular dataset (e.g., sequencing depths and general prevalence of features). Nonetheless, low prevalent microbial features are less likely to be important if they are not found in many of the control or treatment observations. Thus, we consider our method to be a good compromise.

In regards to Fusobacterium nucleatum, we note that the relative abundances of the following taxa were included (i.e., not filtered out by our progressive filtering) in the cirrhosis dataset: 'f_Fusobacteriaceae', 'g_Fusobacterium', 's_Fusobacterium_mortiferum', 's_Fusobacterium_ulcerans', 's_Fusobacterium_nucleatum', 's_Fusobacterium_periodonticum', 's_Fusobacterium_perfoetens'. *Fusobacterium nucleatum* was detected in 73 % of

samples (in 54 % of the healthy individuals and 90 % of cirrhotic ones) and was identified as important by the model and endoR (Figure 5-A, page 8).

The software described in this article is available on Github. Are there plans to submit the R package to the Bioconductor repository?

We submitted the endoR R package to CRAN. The package should be publicly available on CRAN in the near future.

Also, the existing vignettes are rather short and do not include many details. Can those be expanded for better usability of the software?

We agree with the reviewer that the vignettes can be expanded to provide more clarification on the usage of endoR. We have done so by adding additional details and examples in the vignettes (see https://github.com/leylabmpi/endoR/tree/main/vignettes).

Was the creation of the artificial phenotype repeated several times?

Yes. We randomly drew 19 taxa with a prevalence > 50 % and created a fixed set of rules combining thresholds on relative abundances of each taxon (the set of 149 rules is given in Supplementary Table 2). For each simulated phenotype, we randomly picked 3 rules from the set (one for each group) and randomly assigned samples to groups. If samples followed the group rule, their phenotype was set to 1 or otherwise set to -1.

Hence, each artificial phenotype corresponds to a unique combination of interactions for different sets of grouped samples. The process was repeated 51 times for evaluating endoR, resulting in the 51 artificial phenotypes. For the artificial phenotype in Figure 2, we added complexity by using 2 alternative rules in group c, one based on the interaction of 2 taxa and the other based on the relative abundance of 1 taxon (Figure 2 C-D, page 4).

We updated the Methods to clarify the repetition of the simulation procedure.

It seems that the fully simulated data were generated with 100 independent repetitions, but I could not find this information for the artificial phenotypes.

Yes, FSDs were generated with 100 independent repetitions and the APs with 51 (less for this latter assessment due to computational resource constraints). We have now also clarified this in the updated manuscript.

The references to Figure 4 on page 7 are probably referring to Figure 5 and should be adjusted

We thank the reviewer for identifying this error. We have corrected the error.

The asterisk glyphs in Figure 6E are explained in the figure legend but could also be explained in a visual figure legend (such as in Figure 6C).

We thank the reviewer for the helpful suggestion; we have added the asterisk glyph in the legend.

Reviewer #2:

Major comments

In this manuscript, the authors developed an algorithm for interpreting the results from those tree-based machine learning algorithms. Tree-based ML methods have been widely used for microbiome-based prediction due to the complex characteristics of microbiome data and potential high-order interactions among the taxa. However, these ML methods often did not provide sufficient interpretation. Thus, the work is considered to be very significant. The manuscript also reads well and the experiments are carefully designed to demonstrate the method. I have some comments the authors may consider in revision.

We thank the reviewer for their assessments of our work. We have responded to each detailed comment below.

1. The interaction importance defined as the square root of the product of individual feature importance did not seem to conform to the traditional statistical interaction, which describes the phenomenon that the effect of one explanatory variable on the outcome depends on another explanatory variable or the joint effect of two explanatory variables is different from the sum of individual effects. Given the current definition, I do not see how this is related to the traditional statistical interaction. Please elaborate.

Thank you, this is indeed a very important question and we believe one of the key insights into our method. Our intuition behind the decision-wise interaction importance is best illustrated by a simple example:

Consider the decision D with rule “X1>0 & X2>0” and prediction “Y”. From the classical statistical standpoint the interaction between “X1>0” and “X2>0” is important if the same prediction can not be achieved by using only “X1>0” or only “X2>0”. To assess this, we look at the following two modified decisions:

1) **Drm1: rule “X1>0 & X2>0” and prediction “Y1” (where Y1 is based on rule “X2>0”)**
2) **Drm2: rule “X1>0 & X2>0” and prediction “Y2” (where Y2 is based on rule “X1>0”)**

Drm1 corresponds to the best prediction (on the same subselection of the data “X1>0 & X2>0”) that a decision only using “X2>0” can achieve. Similarly, Drm2 corresponds to the best prediction (on the same subselection of the data “X1>0 & X2>0”) that a decision only using “X1>0” can achieve. We can now compare the predictive performance of the different rules, which is done by looking at the decision-wise feature importance. In other words, if Drm1 is better than D, the decision-wise importance of X1

(d1) is large, while if Drm2 is better than D, the decision-wise feature importance of X2 (d2) is large. Now we can consider the following combinations:

1. **"*d1 large and d2 large*": This indicates that there is an interaction effect because dropping either variable leads to a decrease in performance. And indeed the decision-wise feature interaction will be large.**
2. **"*d1 small and d2 large*": This indicates that only "X2>0" is important implying that there is at most a small interaction effect. In this case the decision-wise feature interaction will be small.**
3. **"*d1 large and d2 small*": This indicates that only "X1>0" is important implying that there is at most a small interaction effect. In this case the decision-wise feature interaction will be small.**
4. **"*d1 small and d2 small*": Neither "X1>0" nor "X2>0" are important in this case and therefore the interaction effect should also be small.**

Note that the square root additionally ensures that the feature and interaction importances remain on the same scale.

To clarify this point, we have added a new section entitled "Rationale for the construction of the decision-wise interaction importance" in the Supplementary Methods, page 21.

2. How does the algorithm perform under small sample sizes? In the real world, the majority of microbiome studies have small sample sizes. Performance under small sample sizes should be studied and caveat should be given if the algorithm does not work well for small sample sizes.

The effect of dataset sample size was evaluated in the manuscript, in the section "Evaluation of endoR on simulated data", in the paragraph "endoR improves with the accuracy of fitted models" and shown in Figure 3-E,F (page 6). As stated in the manuscript (paragraph "endoR improves with the accuracy of fitted models" on pages 5 and 6), we found that endoR performed well even on small datasets with N = 200 samples. On such small datasets, endoR captures simple signals (lower recall on Figure 3-F, page 6). Nonetheless, given that microbiome datasets are increasing in size and are sometimes combined (e.g., in meta-analyses), it is nowadays common to conduct analyses on datasets comprising > 200 samples (Almeida et al. 2021; Wu et al. 2020; Pasolli et al. 2017; Zhang et al. 2021; Lee et al. 2022; Duvallet et al. 2017). Since endoR performs well on such sample sizes, we are confident that our method is useful for real-world datasets.

3. Global null conditions should also be studied. Given that there is no relationship between the microbiome and the phenotype, what is the frequency the algorithm will return any findings, and how many false positives if making any findings?

We generated a global null model on the artificial phenotype shown in Figure 2 (page 4) by randomizing the target within each group. Thereby, the sample structure

would be conserved, and the possible target imbalance would be retained within each group (i.e., samples in group c were more likely to have the target label 1). The target should thus be completely de-correlated from the taxa, although the group structure and target balance would be retained. We then used 10x CV to pick the best model consisting of feature selection and fitting of a random forest classifier. The feature selection of the best model (expected accuracy = 56.27 %, with FS parameter gamma = 1 and classifier number of trees = 500) was very stringent, with only 4 features selected. Therefore, we decided to also fit a slightly less accurate model that had a less stringent feature selection step (expected accuracy = 55.54 %, FS gamma parameter = 0.35 and RF number of trees = 100), resulting in 44 selected features. In both cases, endoR identified only 1 stable decision, which corresponds to "samples in group c are more likely to have the target label 1" (due to the imbalance) with a high error of 0.48. Our method was thus able to eliminate noise from the stable decision ensemble and not to return any false positives.

We have added the global null model to the Results, last paragraph of the section "Interpreting tree ensemble models with endoR" (page 3), and we have updated the Methods accordingly (page 15).

In addition, we generated independent global null models for 10 of the artificial phenotypes used to evaluate parameters. Similarly, we performed FS with small gamma values to select 130.8 ± 29.83 features on average, and we fitted the RF classifier with 100 trees; models showed an average expected accuracy of 0.59 ± 0.05. endoR was applied to the 10 models: no stable decision ensemble was found for 6 of 10 models, a decision ensemble made of a unique decision was found for 3 of 10 models, and a decision ensemble comprising 9 decisions was found for 1 of 10 models. The latter case had the highest RF accuracy (0.671), although there was no association between the RF accuracy and number of stable decisions (see Supplementary Figure 2 on page 31).

4. In the analysis of *Methanobacteriaceae* phenotype, how to address the compositional effects? Since the sequencing data are compositional in nature, the increase in the abundance of one taxon will naturally lead to decreases in the relative abundances of other taxa so there is a natural negative influence on the relative abundance of *Methanobacteriaceae*.

Our method does not explicitly take into account the compositional nature of the data. We have added a remark to help clarify: "Nonetheless, endoR does not explicitly take into account the compositional nature of the data inherent to sequencing data." to the Results paragraph "Interpreting tree ensemble models with endoR", line 13 on page 2.

In some recent research (Gordon-Rodriguez, Quinn, and Cunningham 2021; Quinn et al. 2020), it has also been shown that for many microbiome applications, current methods that explicitly take into account the compositionality often do not significantly outperform methods that ignore it. We nevertheless believe that it would be interesting to investigate this further and potentially adapt endoR to account for compositionality. However, such an investigation is outside the scope of the present work.

5. The bootstrapping the authors describes seems to be the sub-sampling technique, not sampling with replacement.

Our approach involved subsampling with replacement, and is thus bootstrapping. We have clarified the Methods accordingly: we added the sentence “Bootstrapping is performed by sub-sampling with replacement.” to the paragraph “Decision ensemble stability”, on page 14.

6. It does not make much sense to compare to those network inference algorithms such as sparCC and gLasso since they are detecting different networks.

We agree that sparCC, gLASSO, and pairwise correlations test different hypotheses. Nonetheless, these are the methods commonly used with similar goals in mind. For instance, to find bacterial interactions occurring in diseased versus healthy individuals, one could generate the interaction networks within each group of individuals using sparCC or gLASSO and compare the networks. Alternatively, one could fit a random forest to predict the health status of individuals and then interpret it with endoR to access interactions predictive of (and associated with) healthy or diseased states.

Upon suggestion from Reviewer #1, we have added linear models with LASSO penalty that are also commonly used in microbiome science and are more comparable to tree-ensemble classifiers (see Figure 4, page 7).

Reviewer #3

Major comments

The paper by Albane Ruaud et al. presents endoR, a machine learning strategy aiming at interpreting a fitted tree ensemble model. The method is validated experimentally on both simulated and real metagenomic data. Results show that endoR is able to infer true associations with more or comparable accuracy than other state-of-the-art approaches while easing model interpretation. The software is available as an open-source R-package on GitHub (https://github.com/leylabmpi/endoR).

This is a nice contribution to the microbiome community. The manuscript is well written and structured.

We thank the reviewer for the supportive comments.

Minor comments

- Is the method suitable for (shotgun) metagenomic data only or could be adopted also for 16S data? Please specify better this aspect in the text.

Our method is suitable for any type of data: shotgun metagenomes (as assessed in our work), 16S rRNA amplicons, or any species counts (e.g., counting of cells via FISH), together with any type of covariate (e.g., sex, age, or diet). This flexibility is a result of utilizing tree ensemble models, which can be applied to any data type in a structured dataset.

We now discuss this topic in the Results, first paragraph of the section "Interpreting tree ensemble models with endoR" (page 2).

- I understand that data used for the analysis are too big to be hosted in github. Could you please put them somewhere else?

We made all data used for analysis publicly available on FigShare: https://figshare.com/projects/Ruaud2022_endoR/142265. The link to datasets was accordingly added to the "Availability of Data and Material" section.

Have the authors made all data and (if applicable) computational code underlying the findings in their manuscript fully available?

Yes, as stated in the "Availability of Data and Material" section, all code is available on GitHub:

- **for the analysis: https://github.com/aruaud/endoR_data_analysis**
- **R-package: https://github.com/leylabmpi/endoR.**

Based on the reviewer's suggestion, we also made datasets publicly available on FigShare: https://figshare.com/projects/Ruaud2022_endoR/142265.

Summary

The PLOS Data policy requires authors to make all data and code underlying the findings described in their manuscript fully available without restriction, with rare exception (please refer to the Data Availability Statement in the manuscript PDF file). The data and code should be provided as part of the manuscript or its supporting information, or deposited to a public repository. For example, in addition to summary statistics, the data points behind means, medians and variance measures should be available. If there are restrictions on publicly sharing data or code —e.g. participant privacy or use of data from a third party—those must be specified.

Reviewer #1: Yes

Reviewer #2: None

Reviewer #3: No: Please make data used for this analysis public available

PLOS authors have the option to publish the peer review history of their article (what does this mean?). If published, this will include your full peer review and any attached files.

If you choose "no", your identity will remain anonymous but your review may still be made public.

Do you want your identity to be public for this peer review? For information about this choice, including consent withdrawal, please see our Privacy Policy.

Reviewer #1: No

Reviewer #2: No

Reviewer #3: No

References

Almeida, Alexandre, Stephen Nayfach, Miguel Boland, Francesco Strozzi, Martin Beracochea, Zhou Jason Shi, Katherine S. Pollard, et al. 2021. "A Unified Catalog of 204,938 Reference Genomes from the Human Gut Microbiome." *Nature Biotechnology* 39 (1): 105–14.

Duvallet, Claire, Sean M. Gibbons, Thomas Gurry, Rafael A. Irizarry, and Eric J. Alm. 2017. "Meta-Analysis of Gut Microbiome Studies Identifies Disease-Specific and Shared Responses." *Nature Communications* 8 (1). https://doi.org/10.1038/s41467-017-01973-8.

Gordon-Rodriguez, Elliott, Thomas P. Quinn, and John P. Cunningham. 2021. "Learning Sparse Log-Ratios for High-Throughput Sequencing Data." *Bioinformatics* , September. https://doi.org/10.1093/bioinformatics/btab645.

Lee, Karla A., Andrew Maltez Thomas, Laura A. Bolte, Johannes R. Björk, Laura Kist de Ruijter, Federica Armanini, Francesco Asnicar, et al. 2022. "Cross-Cohort Gut Microbiome Associations with Immune Checkpoint Inhibitor Response in Advanced Melanoma." *Nature Medicine* 28 (3): 535–44.

Marcos-Zambrano, Laura Judith, Kanita Karaduzovic-Hadziabdic, Tatjana Loncar Turukalo, Piotr Przymus, Vladimir Trajkovik, Oliver Aasmets, Magali Berland, et al. 2021. "Applications of Machine Learning in Human Microbiome Studies: A Review on Feature Selection, Biomarker Identification, Disease Prediction and Treatment." *Frontiers in Microbiology* 12 (February): 634511.

Nearing, Jacob T., Gavin M. Douglas, Molly G. Hayes, Jocelyn MacDonald, Dhwani K. Desai, Nicole Allward, Casey M. A. Jones, et al. 2022. "Microbiome Differential Abundance Methods Produce Different Results across 38 Datasets." *Nature Communications* 13 (1): 342.

Neu, Alexander T., Eric E. Allen, and Kaustuv Roy. 2021. "Defining and Quantifying the Core

Microbiome: Challenges and Prospects.” *Proceedings of the National Academy of Sciences of the United States of America* 118 (51). https://doi.org/10.1073/pnas.2104429118.
Pasolli, Edoardo, Lucas Schiffer, Paolo Manghi, Audrey Renson, Valerie Obenchain, Duy Tin Truong, Francesco Beghini, et al. 2017. “Accessible, Curated Metagenomic Data through ExperimentHub.” *Nature Methods* 14 (October): 1023.
Quinn, Thomas P., Dang Nguyen, Santu Rana, Sunil Gupta, and Svetha Venkatesh. 2020. “DeepCoDA: Personalized Interpretability for Compositional Health Data,” June. https://doi.org/10.48550/arXiv.2006.01392.
Schloss, Patrick D. 2020. “Removal of Rare Amplicon Sequence Variants from 16S rRNA Gene Sequence Surveys Biases the Interpretation of Community Structure Data.” *bioRxiv*. bioRxiv. https://doi.org/10.1101/2020.12.11.422279.
Topçuoğlu, Begüm D., Nicholas A. Lesniak, Mack T. Ruffin 4th, Jenna Wiens, and Patrick D. Schloss. 2020. “A Framework for Effective Application of Machine Learning to Microbiome-Based Classification Problems.” *mBio* 11 (3). https://doi.org/10.1128/mBio.00434-20.
Vangay, Pajau, Benjamin M. Hillmann, and Dan Knights. 2019. “Microbiome Learning Repo (ML Repo): A Public Repository of Microbiome Regression and Classification Tasks.” *GigaScience* 8 (5). https://doi.org/10.1093/gigascience/giz042.
Weiss, Sophie J., Zhenjiang Xu, Amnon Amir, Shyamal Peddada, Kyle Bittinger, Antonio Gonzalez, Catherine Lozupone, et al. 2015. “Effects of Library Size Variance, Sparsity, and Compositionality on the Analysis of Microbiome Data.” *PeerJ*. https://doi.org/10.7287/peerj.preprints.1157v1.
Wu, Sicheng, Chuqing Sun, Yanze Li, Teng Wang, Longhao Jia, Senying Lai, Yaling Yang, et al. 2020. “GMrepo: A Database of Curated and Consistently Annotated Human Gut Metagenomes.” *Nucleic Acids Research* 48 (D1): D545–53.
Zhang, Qingfeng, Kai Yu, Shihua Li, Xiaolong Zhang, Qi Zhao, Xia Zhao, Zekun Liu, Han Cheng, Ze-Xian Liu, and Xiaoxing Li. 2021. “gutMEGA: A Database of the Human Gut MEtaGenome Atlas.” *Briefings in Bioinformatics* 22 (3). https://doi.org/10.1093/bib/bbaa082.
Zhou, Yi-Hui, and Paul Gallins. 2019. “A Review and Tutorial of Machine Learning Methods for Microbiome Host Trait Prediction.” *Frontiers in Genetics* 10 (June): 579.
