# Supplementary material for: Interpreting tree ensemble machine learning models with endoR
Source: PLoS Comput Biol. 2022 Dec 14;18(12):e1010714. doi: 10.1371/journal.pcbi.1010714 (PMC9797088; doi:10.1371/journal.pcbi.1010714)
Supplement: Attachment — Submitted filename: Response_to_reviewers.pdf [file pcbi.1010714.s037.pdf]

Dear Dr. Youngblut,

Thank you very much for submitting your manuscript "Interpreting tree ensemble machine learning models with endoR" for consideration at PLOS Computational Biology. As with all papers reviewed by the journal, your manuscript was reviewed by members of the editorial board and by several independent reviewers. The reviewers appreciated the attention to an important topic. Based on the reviews, we are likely to accept this manuscript for publication, providing that you modify the manuscript according to the review recommendations.

We believe that major issues have been addressed and that the outstanding doubts of Reviewer #1 are quickly addressable.

Please prepare and submit your revised manuscript within 30 days. If you anticipate any delay, please let us know the expected resubmission date by replying to this email.

When you are ready to resubmit, please upload the following:

[1] A letter containing a detailed list of your responses to all review comments, and a description of the changes you have made in the manuscript. Please note while forming your response, if your article is accepted, you may have the opportunity to make the peer review history publicly available. The record will include editor decision letters (with reviews) and your responses to reviewer comments. If eligible, we will contact you to opt in or out

[2] Two versions of the revised manuscript: one with either highlights or tracked changes denoting where the text has been changed; the other a clean version (uploaded as the manuscript file).

Important additional instructions are given below your reviewer comments.

Thank you again for your submission to our journal. We hope that our editorial process has been constructive so far, and we welcome your feedback at any time. Please don't hesitate to contact us if you have any questions or comments.

Sincerely,

Luis Pedro Coelho

Academic Editor

PLOS Computational Biology

Dina Schneidman

Software Editor

PLOS Computational Biology

****

A link appears below if there are any accompanying review attachments. If you believe any reviews to be missing, please contact ploscompbiol@plos.org immediately:

We believe that major issues have been addressed and that the outstanding doubts of Reviewer #1 are quickly addressable.

Reviewer's Responses to Questions

Comments to the Authors:

Please note here if the review is uploaded as an attachment.

Reviewer #1: I want to thank the authors very much for addressing my previous comments. I can imagine that it was a lot of work to include the LASSO classifier in their comparison as well and I appreciate their effort. Also, I thank the authors to submitting their package to CRAN. I hope it can be useful to the wider community. Lastly, restructuring the paper improved its readability very much.

We thank the reviewer for their thorough review and encouragement!

I would have some question regarding the comparison to other methods, again.

Supplementary Figure 7 and Supplementary Figure 8 EF seem to identical. Is this intended?

We thank the reviewer for spotting this error. We inadvertently used the wrong plots for Supplementary Figure 8EF; we have now replaced them for the FPR and TPR and moved them to Figure 4 (page 13) as suggested hereafter.

Also, I cannot really find correspondence between Figure 4 and Supplementary Figure 8 A-D, although they should show the same data (evaluation on the AP data). I would hope that the authors can clarify my confusion here. For example, the orange dot in SFig 8A indicated that at this FPR (it should be FPR in the axis label as well), only LASSO with interactions and the Wilcoxon perform worse than endoR, but in Fig 4A, Lasso with interaction performs better than the Wilcoxon and normal Lasso performs worse (but has a similar TPR in SFig 8A). From the figure legends I would think that the same data are shown, but as rates instead of numbers.

Thanks for pointing this out: our plots were inconsistent due to the legend labels and the confusion in data for the lasso with interactions curve. We have corrected the figures.

Also, I would suggest to show both rates and the numbers in the main figure. As a reader, I personally would be more interested into the TPR at a given FPR than in absolute numbers for five FP detections.

We have now grouped the plots by data with both the TP/FP numbers and rates, such that Figure 4 now consists of APs (formerly Figure 4 and Supplementary Figure 8 A-B) and Supplementary Figure 7 of FSDs (formerly Supplementary Figure 7 and Supplementary Figure 8 E-F).
The captions were modified accordingly; see page 13 of the main text for Figure 4 and page 6 of the Supplementary Figures for Supplementary Figure 7.

Reviewer #2: The authors have addressed my comments sufficiently.

We thank the reviewer for their helpful feedback, which has improved this manuscript.

Reviewer #3: I feel the authors have answered positively to my previous comments and modified the manuscript according to them.

We thank the reviewer for their helpful feedback, which has improved this manuscript.
